# Quantifying the optimal strategy of population control of quorum sensing network in *Escherichia coli*

**DOI:** 10.1038/s41540-021-00196-4

**Published:** 2021-09-02

**Authors:** Xiang Li, Jun Jin, Xiaocui Zhang, Fei Xu, Jinjin Zhong, Zhiyong Yin, Hong Qi, Zhaoshou Wang, Jianwei Shuai

**Affiliations:** 1 Department of Physics, College of Physical Science and Technology, Xiamen University, Xiamen, China; 2 State Key Laboratory of Cellular Stress Biology, Innovation Center for Cell Signaling Network, Xiamen University, Xiamen, China; 3 National Institute for Data Science in Health and Medicine, Xiamen University, Xiamen, China; 4 Complex Systems Research Center, Shanxi University, Shanxi Taiyuan, China; 5 Institute of Biochemical Engineering, Department of Chemical and Biochemical Engineering, College of Chemistry and Chemical Engineering, Xiamen University, Xiamen, China; 6 The Key Lab for Synthetic Biotechnology of Xiamen City, Xiamen University, Xiamen, China

**Keywords:** Dynamic networks, Microbiology

## Abstract

Biological functions of bacteria can be regulated by monitoring their own population density induced by the quorum sensing system. However, quantitative insight into the system’s dynamics and regulatory mechanism remain challenging. Here, we construct a comprehensive mathematical model of the synthetic quorum sensing circuit that controls population density in *Escherichia coli*. Simulations agree well with experimental results obtained under different ribosome-binding site (RBS) efficiencies. We present a quantitative description of the component dynamics and show how the components respond to isopropyl-β-D-1-thiogalactopyranoside (IPTG) induction. The optimal IPTG-induction range for efficiently controlling population density is quantified. The controllable area of population density by acyl-homoserine lactone (AHL) permeability is quantified as well, indicating that high AHL permeability should be treated with a high dose of IPTG, while low AHL permeability should be induced with low dose for efficiently controlling. Unexpectedly, an oscillatory behavior of the growth curve is observed with proper RBS-binding strengths and the oscillation is greatly restricted by the bacterial death induced by toxic metabolic by-products. Moreover, we identify that the mechanism underlying the emergence of oscillation is determined by the negative feedback loop structure within the signaling. Bifurcation analysis and landscape theory are further employed to study the stochastic dynamic and global stability of the system, revealing two faces of toxic metabolic by-products in controlling oscillatory behavior. Overall, our study presents a quantitative basis for understanding and new insights into the control mechanism of quorum sensing system, providing possible clues to guide the development of more rational control strategy.

## Introduction

Quorum sensing refers to the phenomenon that bacteria can sense their population density and regulate gene expression using chemical signaling molecules, thereby promoting cell-to-cell communication to synchronize the behaviors of bacteria, such as bioluminescence, biofilm formation and maturation, virulence-factor expression, motility, and so on^1–3^. The signaling molecules that were secreted by bacteria can regulate their own biological behavior, and are called autoinducers. Autoinducers will regulate the expression of target genes once their concentration reaches a certain threshold. Bacterial quorum sensing system can be generally divided into two main types: Gram-negative and Gram-positive of the types of bacteria^4,5^. Acyl-homoserine lactones (AHLs) and peptides are included in the major types of quorum sensing signals. Gram-negative bacteria use fatty acid derivatives as signaling molecules, which mostly belong to the class of AHLs in the quorum sensing system^6^. Acyl-homoserine lactone (AHL) is the autoinducer that activates the transcription factor to regulate luminescence in *Vibrio fischeri*^7^. Unlike Gram-negative bacteria, a small peptide (AIP) secreted through ATP-binding cassette transporters acts as the signaling molecules in Gram-positive bacteria^8^. The sensor kinases in the two-component system can sense AIP and then trigger a series of phosphorylation events, leading to relative gene expression. Autoinducer-2 (AI-2), a kind of signaling molecule that can participate in the quorum sensing system of both Gram-negative and Gram-positive bacteria, is believed to be a general emissary for facilitating interspecies communication^9^. To better understand the quorum sensing system control mechanisms, an increasing number of studies have artificially synthesized the quorum sensing system in marine bacteria and further imported it into *E. coli* bacteria^10,11^.

Mathematical modeling is a powerful approach to study the regulatory mechanisms of biological systems^12,13^. To explore the dynamic behaviors of bacterial quorum sensing, a great number of theoretical models have been established^14–16^. You et al. imported two fragments of luxI/luxR genes in *E. coli* and built a quorum sensing system model to explore the relation between AHL and lethal protein^17^. Li et al. artificially synthesized a bacterial quorum sensing system of AI-2 signaling and constructed a stochastic model to study the hierarchical organization of luxS-derived AI-2 circuitry in *E. coli*, suggesting that the stochastic dynamics can be linked to cell physiology^10^. Chen et al. developed a model of synthetic gene network by using the luxI–luxR quorum sensing system to analyze the multicellular system dynamics in *Vibrio fischeri*^18^ confirming that noises are essential for inducing the system cooperative behaviors. Torres-Cerna et al. recently proposed a model to capture the AI-2 dynamics on *E.coli* quorum sensing, highlighting the dependency of the extracellular AI-2 activity and the lsr operon expression on the cell growth^19^.

Although these models have successfully dissected many aspects of dynamic behaviors of the quorum sensing system, development of quantitative mathematical models for precisely studying the control mechanisms of the system is still a major challenge^20^. To subtly control bacterial cell density, Wang et al. built a synthetic cell–cell communication system that combined the cell death BioBricks and quorum sensing mechanism in *Vibrio fischeri*^21^. Even though a simple abstract model has been employed previously^21^, systematical analysis of the component dynamics cannot be performed as well as unraveled new mechanism insights into the system. Based on the experimental data by Wang et al., we develop a mathematical signaling network model that is incorporating the modules of gene transcription, AHL synthesis processes, extracellular AHL_o_, tetramer LuxR_2_:AHL_2_ formation, and CcdB-induced bacterial population decline. We explore the dynamic responses of the components and attempt to quantify the optimal isopropyl-β-D-1-thiogalactopyranoside (IPTG) induction dose for efficient population control. Moreover, whether and how the population density can be regulated by AHL permeability is studied. An oscillatory behavior of the growth curve is predicted within a proper ribosome-binding site (RBS) binding-strength range. To capture the underlying oscillatory mechanism, we further determine that the toxic metabolic coefficient largely limited the oscillation and quantify the corresponding controllable ranges. The underlying mechanism of the emergence of oscillation is identified to be determined by the negative feedback loop structure in the quorum sensing system. Ultimately, both the bifurcation analysis and landscape theory are utilized to quantify the roles of the key reactions and toxic factors in controlling bacterial density. Taken together, our study provides a quantitative description of the quorum sensing system control mechanism and unveils several new insights into the system.

## Results

### IPTG-induced dynamic response of the components determines bacteria-growth curve

**Fig. 1:**
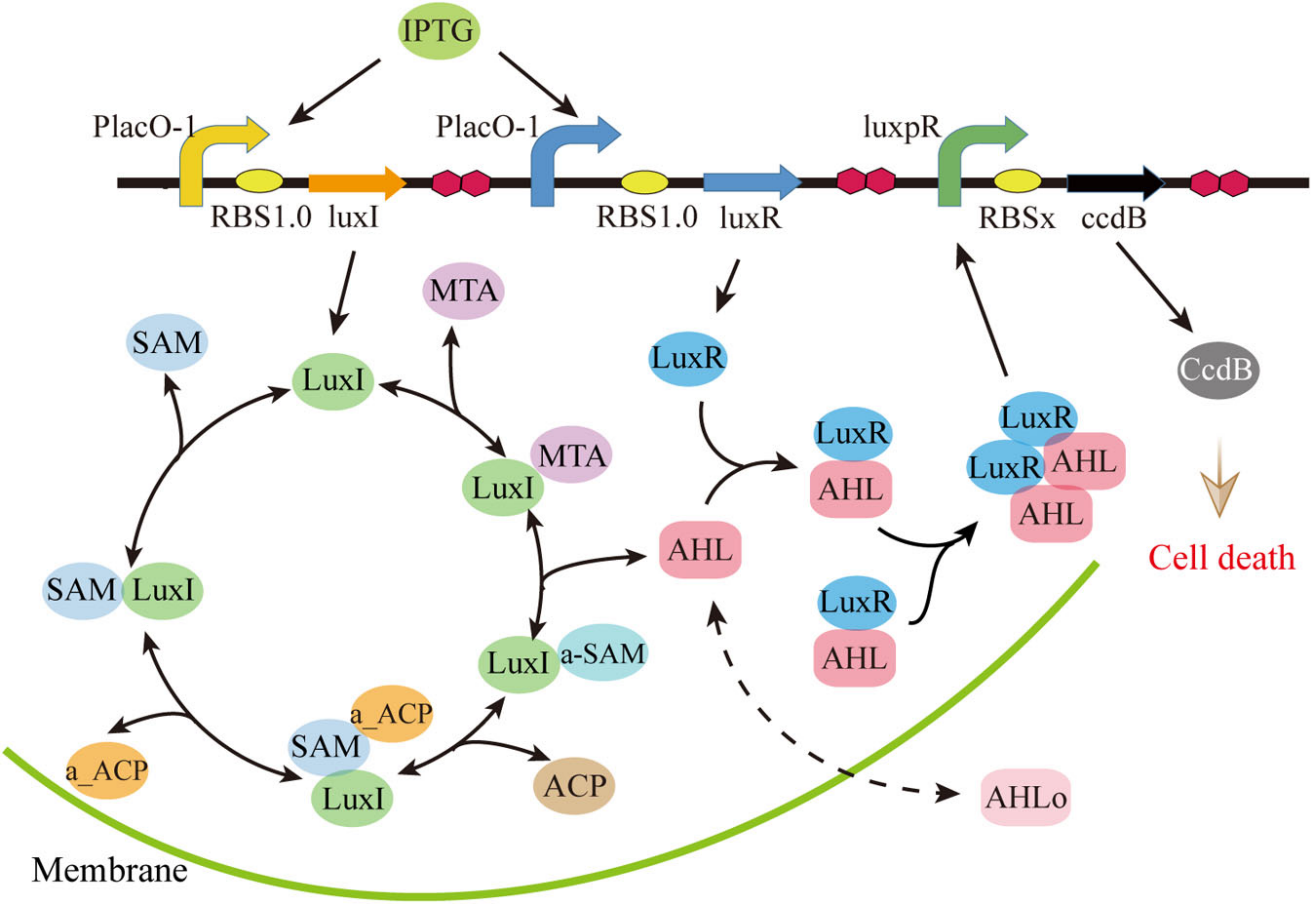
Schematic representation of the synthetic quorum sensing signaling that controls bacterial population density. The subscript “x” in RBSx represents different RBSs, such as RBS_0.07_, RBS_0.3_, and RBS_1.0_.

The synthetic quorum sensing signaling in *E. coli* is shown in Fig. 1. Once the inducer isopropyl β-D-l-thiogalactopyranoside (IPTG) is added to a culture, it will act on the promoter *P*_lacO-1_ to trigger the expression of *luxI* and *luxR* genes, thereby generating proteins LuxI and LuxR. LuxI protein induces the synthesis of signaling molecule AHL^22^. As the left circle presented, LuxI protein uses SAM and acyl-ACP as substrates to synthesize AHL^23^. The homoserine lactone part of AHL is derived from *S*-adenosyl methionine (SAM) and the *N*-acyl side chain of AHL is provided by acyl-ACP or acyl-CoA. SAM is converted into methyl-thio-adenosine (MTA) and AHL by LuxI. LuxR binds to AHL to form the LuxR:AHL complex. Then two LuxR:AHL complexes combine together to form the active tetramer complex, LuxR_2_:AHL_2_^24,25^, which will subsequently bind to the promoter *luxpR* and induce the expression of the lethal gene to produce the protein CcdB^26,27^. In the quorum sensing systems, CcdB is completely artificial, which can inactivate DNA gyrase and kill the host bacteria, thereby regulating bacterial population density^28,29^.

**Fig. 2:**
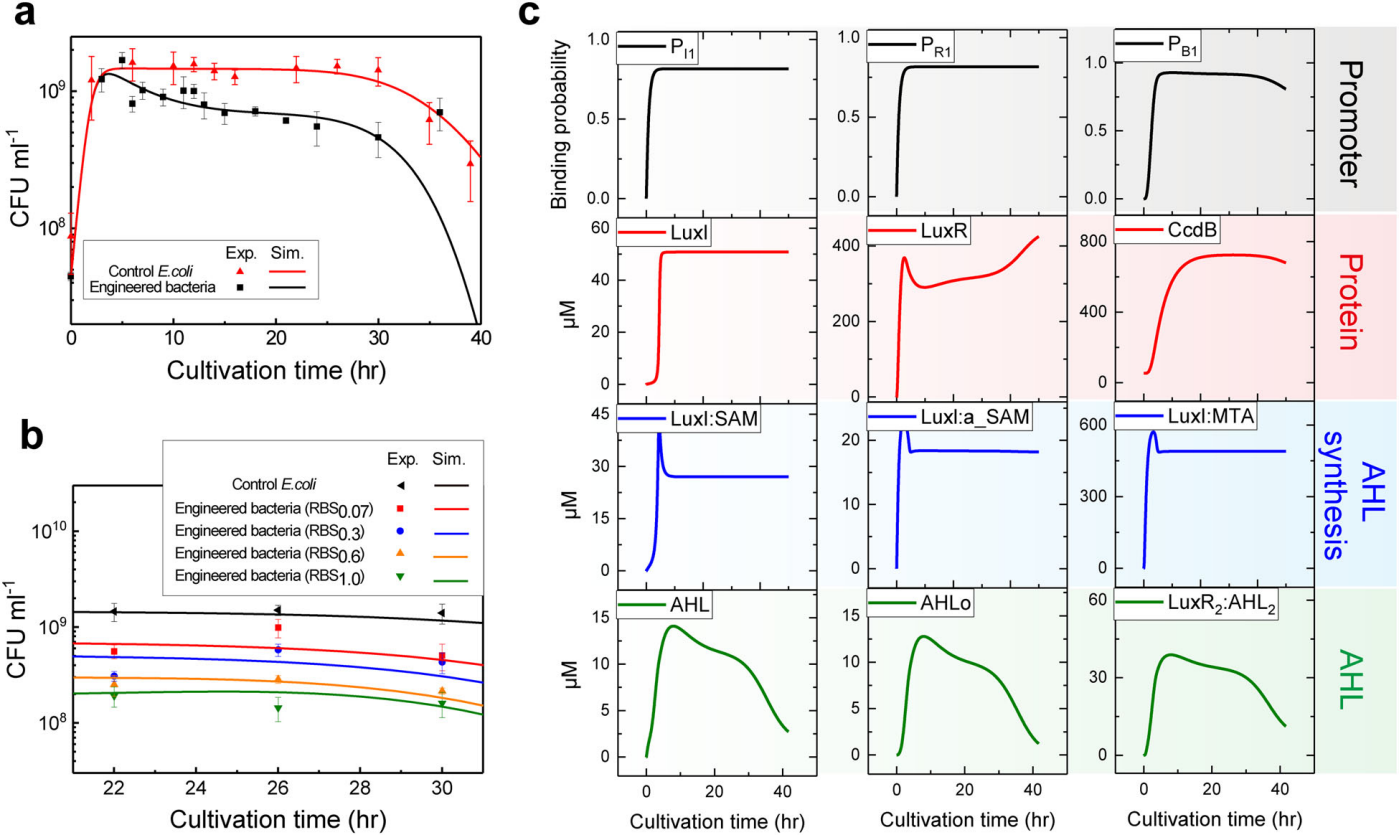
Dynamic responses of the key components in the model upon IPTG induction. **a** Comparison of population density for experimental data and simulation results in control and engineered (RBS_0.07_) *E. coli*. **b** Test of model prediction of the effect of increasing RBS efficiency on population density. Symbols and lines are experimental data and simulation results, respectively. The experimental data are from a previous publication^21^. **c** Time courses of the model’s four major modules, including the promoters responding (gray background), corresponding protein expression (red background), components relative to AHL synthesis (blue background), and components containing AHL (green background). The error bars represent the standard deviation of at least three replicates.

The proposed model is developed based on the layout displayed in Fig. 1, containing the modules of IPTG-induced gene transcription of LuxI and LuxR, LuxI-dependent AHL synthesis, LuxR_2_:AHL_2_ induced the production of CcdB, and the dynamics of bacterial population *N*. We determine the model parameters by fitting the experimental bacteria-growth curves of wild-type *E. coli* and the engineered bacteria with RBS_0.07_ *E. coli*. IPTG is added at the beginning, which is consistent with the experiment^21^. As shown in Fig. 2a, the model can well fit the corresponding growth curves, including the log phase, stationary phase, and decline phase of the bacterial population density^30,31^. During the log phase, the culture medium is rich in nutrients and there is barely accumulation of metabolic by-products. The bacteria grow rapidly and the population density shows a logarithmic increase. Log phase cannot continue indefinitely due to the consumption of nutrients. When the bacterial population density is beyond a certain range, the nutrients are no longer sufficient, and the accumulation of metabolic by-products has reached a certain level, which will kill the bacteria. Stationary phase results from a situation in which the populations of new bacteria and dead bacteria present a dynamic equilibrium and thus the density is in a stable period. This could be caused by the lack of nutrients, environmental temperature above or below the tolerance band for the species, or other injurious conditions. During the later period, nutrients become lacking and toxic metabolic by-products are accumulated massively. The population of dead bacteria far exceeds new bacteria, leading to the density of bacteria decreases rapidly and enters the decline phase. However, the engineered strain-density data of the decline phase are not well fitted (black line in Fig. 2a). This is because of the simplified consideration of the effect of toxic metabolic by-products on cell death. Yet the stationary phase of the density, which was fitted nicely and predicted by our model (Fig. 2a, b), is the main focus in our study.

Wang et al. also investigated different efficiency of RBSx to regulate the expression of lethal protein genes in their study^21^, indicating that RBS_0.3_, RBS_0.6_, and RBS_1.0_ can subtly control bacterial population density at different levels. To test the validity of our model, we therefore employ the model to simulate the dynamic behavior of bacterial growth curves with the different RBS efficiencies. Consistent with the experimental data, our model well reproduces the result that increasing RBS efficiency decreases the population density in the stationary phase (Fig. 2b).

To quantitatively reflect the dynamical behaviors of the synthetic quorum sensing network, we present an overview of the kinetics of key components in response to IPTG induction (Fig. 2c). The probabilities of the promoters of *luxI* gene and *luxR* gene responding to the inducer have the same change process (Fig. 2c, gray background). They both reach stability at about 4 h and then switch to the stationary phase. However, the time for the *ccdB* gene promoter responding to the inducer to reach stability is 1 h later (~5 h) than *luxI* gene and *luxR* gene. This is because the activation of *ccdB* gene promoter is triggered by the tetramer complex LuxR_2_:AHL_2_ that is mediated by LuxI. Activation of *ccdB* gene promoter appears to significantly decrease after 30 h, which is induced by the decrease of its inducer LuxR_2_:AHL_2_ complex during the decline phase (Fig. 2c, green background).

**Fig. 3:**
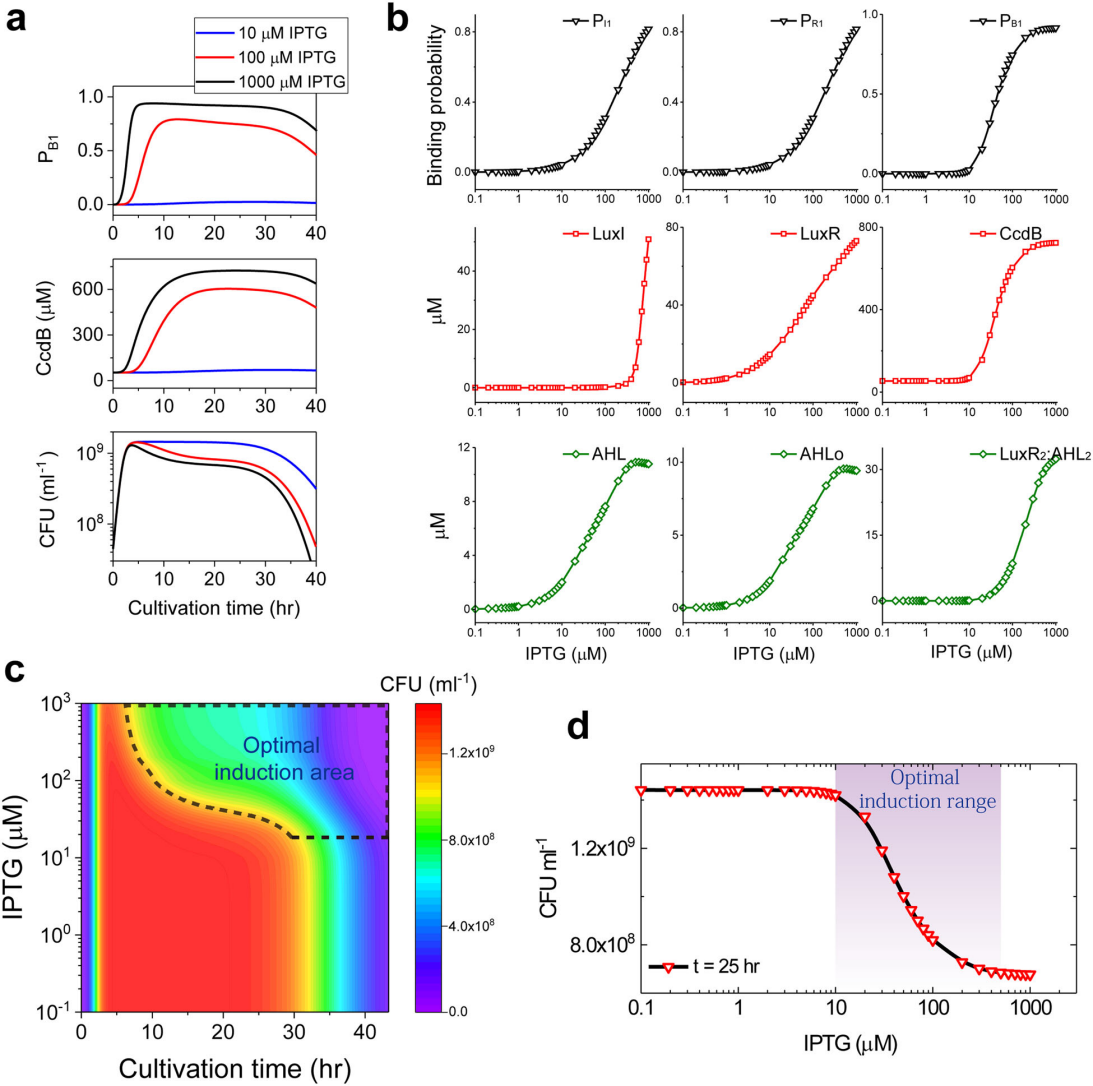
Analysis of the various degrees of IPTG induction on the quorum sensing components activities. **a** Time courses of *ccdB* gene promoter responding probability (P_B1_), CcdB protein, and population density upon three typical IPTG-dose inductions. **b** Dynamic responses of the key components upon a series of doses of IPTG induction during the stationary phase. **c** Change of bacteria-growth curve with different doses of IPTG induction. **d** Dynamic responses of population density upon a series of doses of IPTG induction during the stationary phase.

Dynamic changes of LuxI and LuxR proteins indicated that LuxI protein level is stabilized at about 5 h, while LuxR keeps rising for a long time (Fig. 2c, red background). The CcdB protein level that was determined by the dynamics of *ccdB* gene promoter is stabilized at about 12 h and decreases at about 30 h. The complex LuxI:SAM, LuxI:a_SAM, and the by-product complex LuxI:MTA that involves AHL production are both logarithmic increase, and then decrease and stabilize at a certain level (Fig. 2c, blue background). Simulation results suggest that the behaviors of intracellular and extracellular signaling molecule AHLs are basically the same (Fig. 3c, green background). Both of them rapidly increase and then gradually decrease. The intracellular AHL level is invariably a little higher than the extracellular AHL. The tetramer complex LuxR_2_:AHL_2_ exhibits the similar dynamic behavior as that of AHL, but with a nearly 3-fold higher level. Thus, our model-based analysis presents a quantitative description of the component dynamics of the system after IPTG induction, which can hardly be observed in experiment.

### Optimal induction range of IPTG for controlling bacterial population density

Studies have suggested that IPTG can exacerbate the substrate toxicity and cause damage to *E.coli*^32,33^. When using IPTG to induce the expression of foreign proteins, its level should be as low as possible to achieve the best induction effect^34^. Therefore, finding the optimal induced level of IPTG to control bacterial density is necessary. To further explore the physiological impact of IPTG inducer on the synthetic quorum sensing system, we first present the changes of the key components (the responding probability P_B1_, CcdB, and population density *N*) in response to three typical doses of IPTG induction (10, 100, and 1000 μM). As the simulation results show in Fig. 3a, the three components present a dose-dependent kinetics. For a low dose of IPTG (10 μM), the probability of the *ccdB* gene promoter responding to LuxR_2_:AHL_2_ complex (P_B1_) and CcdB level keeps low and the synthetic system barely shows any response. Thus, the growth curve can hardly be regulated (blue lines). While a high dose of 100 μM triggers the transcription of *ccdB* gene promoter and CcdB production, thereby decreasing the population density during the stationary phase (red lines). With an increase of IPTG dose, the maximal responding probability (P_B1_) and CcdB levels show a continuous increase, leading to the further decrease of population density (black lines).

To comprehensively quantify the regulatory processes, dynamical behaviors of the key components are studied under a series of IPTG induction levels during the stationary phase at *t* = 25 h. Simulation results suggest that components in modules exhibit different threshold responses to IPTG level (Fig. 3b). In the module of gene transcription, the three responding probabilities (P_I1_, P_R1_, and P_B1_) are low at low IPTG level and the three gene promoters are activated and start to produce proteins when IPTG level is greater than 10 μM. Different from the sustained-increase tendency of the promoters responding probabilities P_I1_ and P_R1_, P_B1_ almost reaches saturation at 200 μM, suggesting that the complex LuxR_2_:AHL_2_ level is sufficient to maximize the activation of *ccdB* gene promoter. However, behaviors of the gene-encoded proteins are completely different. LuxI level keeps at a quite low level and rapidly increases when the level of IPTG is greater than 400 μM. While the patterns of LuxR and CcdB proteins are basically consistent with the corresponding gene-responding probabilities. As an effector protein in the system, CcdB presents a switch-like response to IPTG (Fig. 3b), which is required for the optimal control strategy. The switch-like response might be indirectly supported by the experimental observations in three different synthetic *E. coli* systems^35–37^. In the module of AHL synthesis, intracellular and extracellular signaling molecule AHLs show the same dynamical responses, which gradually increases with IPTG at the threshold level of 2 μM IPTG. But the formation of the tetramer complex LuxR_2_:AHL_2_ initializes at a higher threshold level of about 20 μM IPTG. Compared with AHL and AHL_o_, complex AHL_2_:LuxR_2_ shows a steeper change curve with IPTG.

Importantly, the role of IPTG level in controlling bacterial population density is quantified. As the simulation result is shown in Fig. 3c, increase of IPTG induction level does not affect the log phase of bacteria-growth curve, but decreases the population density during the stationary phase when IPTG is above about 10 μM. Besides, the duration of the stationary phase is increased as the increase of IPTG. The shape of the growth curve is barely regulated when IPTG level is lower than 10 μM, indicating a threshold level of IPTG in controlling population density. Therefore, there exists a response-induction range of IPTG for the synthetic quorum sensing circuit. We further plot the variation of bacterial population with IPTG during the stationary phase at *t* = 25 h. It can be clearly seen from Fig. 3d that the sensitivity range of population density to IPTG is between 10 and 500 μM. Lower- (<10 μM) or higher-level (>500 μM) IPTG induction variations can barely regulate population density. Thus, for efficient control, the optimal induced concentration of IPTG should be restricted in the sensitivity range.

### AHL-permeability-regulated population density is IPTG dependent

As the signaling molecule in the synthetic quorum sensing system, AHL can permeate outside the bacterial cells. A number of models have been developed to evaluate the complex role of AHL in various quorum sensing systems^17,26,38^. However, a quantitative analysis of whether and how the permeability of AHL can regulate population density is still lacking. To evaluate their performance, AHLs inside and outside the cells are considered and the permeability is represented by *k*_15_ in our model.

**Fig. 4:**
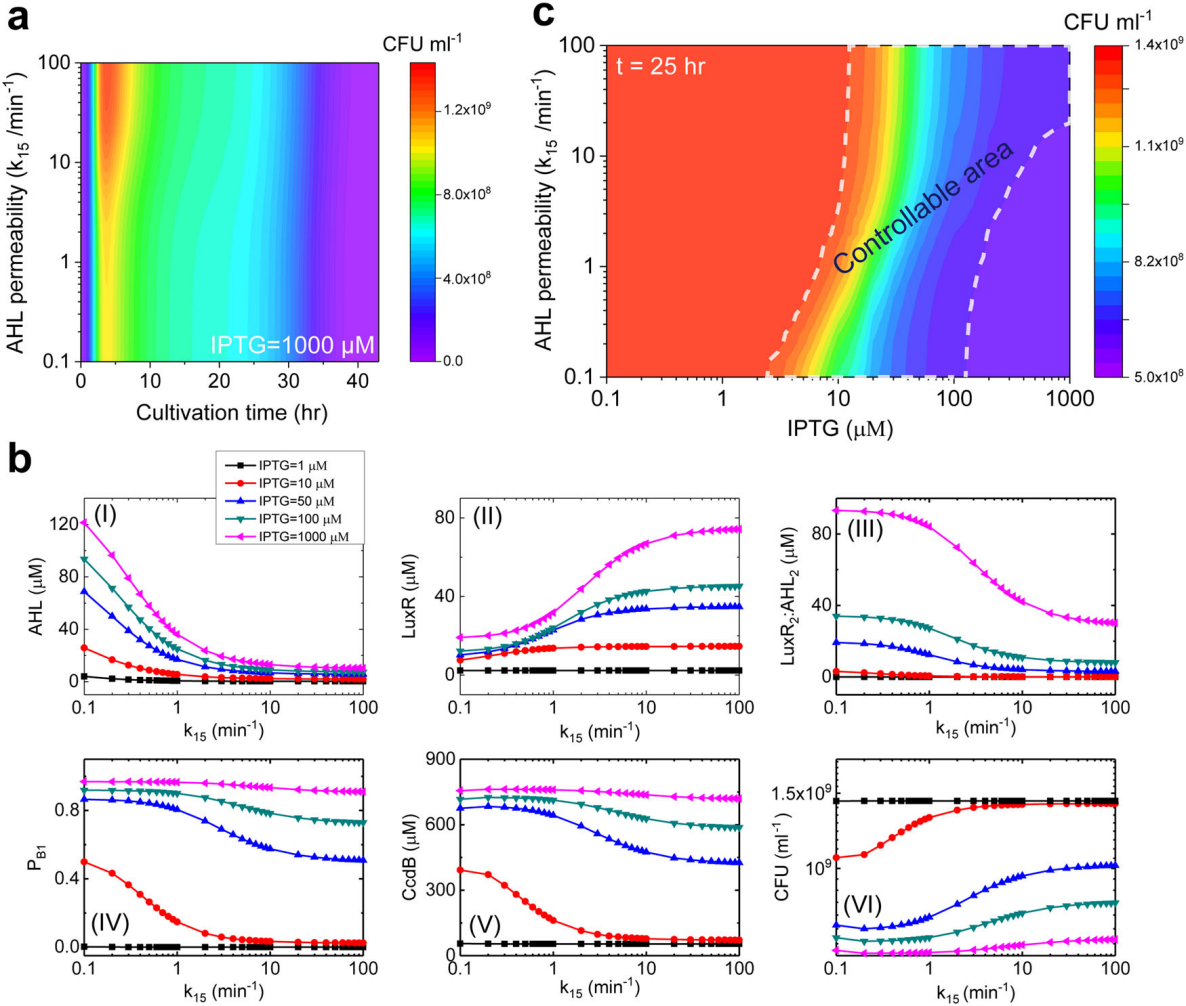
Role of AHL permeability in controlling bacteria population density. **a** Change of bacteria-growth curve under different permeability of AHL (*k*_15_) with 1000 μM IPTG induction. **b** Dynamic responses of the key components under different permeability of AHL with various IPTG induction doses during the stationary phase. **c** Change of population density under different permeability of AHL with a series of IPTG induction doses during the stationary phase.

The effects of AHL permeability on the bacteria-growth curve are studied for the system upon 1000 μM IPTG induction (Fig. 4a). The parameter of AHL permeability (*k*_15_) is considered to vary in a wide reasonable range (0.1 min^−1^ < *k*_15_ < 100 min^−1^) based on the previous literature^39^, which extends three orders around its standard value in the model. As the simulation result suggested, at low permeability of AHL (~0.1 min^−1^ < *k*_15_ < ~1 min^−1^), variations of permeability hardly influence the growth curve. While at the high permeability of AHL (~1 min^−1^ < *k*_15_ < ~100 min^−1^), the increase of permeability raises the maximal amount of cells during the log phase, but barely affects the stationary phase and decline phase of the growth curve. Thus, AHL permeability cannot control population density during the stationary phase at strong IPTG induction.

We next seek to dissect whether AHL permeability can regulate population density upon other induction levels of IPTG. Five typical IPTG induction levels (1, 10, 50, 100, and 1000 μM) are considered to predict the effects of AHL permeability on the key component dynamics during the stationary phase (Fig. 4b). Intracellular AHL shows a gradually increasing response to IPTG increase at a certain AHL permeability (Fig. 4bI). When IPTG induction level is very low (1 μM), the production of AHL is also very low. No matter how high permeability of AHL is, AHL level inside the bacteria is close to zero (black point and line). With high-level IPTG induction, LuxI is generated in a large quantity, and AHL is accumulated inside the bacteria. As AHL permeability increases, a large amount of AHL will flow out of the bacteria, and AHL inside the bacteria will be significantly reduced. When AHL permeability is large enough (*k*_15_ > 20 min^−1^), bacterial membrane is mostly at a fully permeable state. AHL concentrations inside and outside the bacteria are nearly the same. Since the external volume of bacteria is much larger than the internal volume, AHL concentrations both inside and outside are stabilized at a relatively low level.

The response curves of LuxR and complex LuxR_2_:AHL_2_ to AHL permeability variation during the stationary phase are also quantified (Fig. 4bII, III). LuxR and complex LuxR_2_:AHL_2_ levels are close to zero with low IPTG induction (1 μM). Increasing IPTG induction level elevates both the amounts of LuxR and LuxR_2_:AHL_2_. As the results suggested, AHL permeability affects the concentration distributions of free LuxR and the LuxR in complex LuxR_2_:AHL_2_. A low AHL permeability (~0.1 min^−1^ < *k*_15_ < ~1 min^−1^) that induces a high level of AHL in bacteria results in a low concentration of LuxR and a high concentration of LuxR_2_:AHL_2_ in bacteria. While a low level of AHL in bacteria aroused by high AHL permeability (~1 min^−1^ < *k*_15_ < ~100 min^−1^) leads to a high concentration of LuxR and a low concentration of LuxR_2_:AHL_2_.

The responding probability of *ccdB* gene promoter to the inducer LuxR_2_:AHL_2_ is barely affected with the change of AHL permeability upon too low or too high level of IPTG induction (Fig. 4bIV). With low level of IPTG induction (~1 μM), the level of the inducer complex LuxR_2_:AHL_2_ is low and thus the responding probability of *ccdB* gene promoter is small. When IPTG level is high (~1000 μM), the level of the complex LuxR_2_:AHL_2_ is high enough to maximize its responding to the *ccdB* gene promoter. The responding probability tends to 1 with different AHL permeability. When IPTG induction level is at an appropriate value (~10 μM), the responding probability of the *ccdB* gene promoter to LuxR_2_:AHL_2_ becomes small as the permeability increases. This is because the concentration of LuxR_2_:AHL_2_ becomes low. Besides, the results also indicate that a quite low level of LuxR_2_:AHL_2_ is sufficient to induce an ~50% responding probability increase of the *ccdB* gene promoter.

The change of CcdB protein with AHL permeability is the same as that of the *ccdB* promoter responding probability (Fig. 4bV). Since the lethal CcdB protein will kill the host bacteria, the dynamic responses of bacterial population density to AHL permeability are opposite to that of CcdB protein. As the result shows (Fig. 4bVI), an increase of AHL permeability raises the bacterial population density. Besides, the results also reveal that the ranges of AHL permeability for efficiently controlling population density are discrepant. For 10 μM IPTG induction, the controllable range of AHL permeability is corresponding to ~0.2 min^−1^ < *k*_15_ < ~2 min^−1^. While for 50 μM IPTG induction, the controllable range is ~1 min^−1^ < *k*_15_ < ~20 min^−1^. Thus, the controllable range of AHL is dependent on the IPTG induction level. Taken together, we systematically quantify the controllable area of population density by AHL permeability upon different IPTG induction levels. As the controllable area is indicated in Fig. 4c, variation of AHL permeability that can regulate population density should be upon the IPTG-induction range from ~2 to ~100 μM. Upon low-dose IPTG induction, AHL permeability within a low range can control population density. But for high-dose IPTG induction, AHL permeability should be high for efficient controlling.

### Oscillatory behavior of bacterial growth curve determined by RBS efficiency and toxic factors

**Fig. 5:**
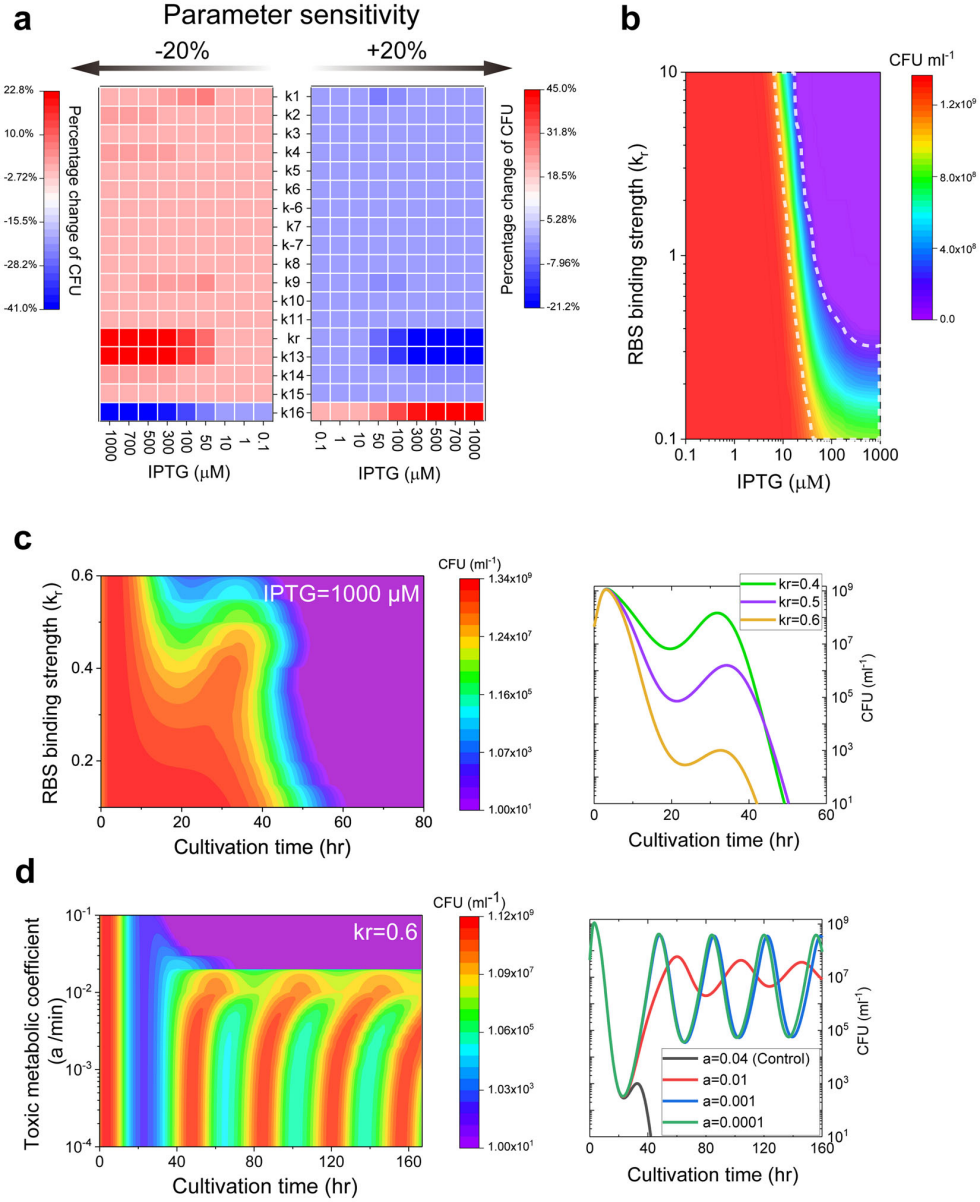
RBS-binding efficiency and toxic metabolic by-products in controlling bacteria population density. **a** Sensitivity analysis of the parameters in mediating population density. Parameters are varied ±20% from their defined values reported in Supplementary Table 1. The corresponding percentage change of the population density is calculated. **b** Change of population density under different RBS-binding strengths (*k*_*r*_) with a series of IPTG induction doses during the stationary phase. **c** Change of bacteria-growth curve under different RBS-binding strengths (*k*_*r*_) with 1000 μM IPTG induction. The growth curves corresponding to the three typical RBS-binding strengths (0.4, 0.5, and 0.6) are shown in the right panel. **d** Change of bacteria-growth curve under different toxic metabolic coefficients (*a*) at the RBS-binding strength of 0.6 with 1000 μM IPTG induction. The growth curves corresponding to the three typical coefficients (0.01, 0.001, and 0.0001) are shown in the right panel.

To identify the biochemical reaction processes that have great effect on the population during the stationary phase, parameter-sensitivity analysis is performed upon different levels of IPTG induction. Single-parameter sensitivity is conducted by varying the key process parameter ±20% from its default value and the corresponding percentage change of the population density (CFU/mL) is calculated accordingly. As shown in Fig. 5a, upon weak IPTG induction (0.1, 1, and 10 μM), population density barely responds to parameters variations, since the synthetic quorum sensing circuit is not triggered (Fig. 3a, blue lines). With the increase of IPTG induction in the range of 50–1000 μM, population density is gradually influenced by the variations of three parameters *k*_*r*_, *k*_13_, and *k*_16_, denoting the RBS-binding strength corresponding to *ccdB* gene, the self-synthesis rate of CcdB by the *ccdB* gene, and the reproducing rate of the bacteria, respectively. But the rest of the parameters show little effects on the population density. The sensitivity analysis further suggests that most of the biochemical reactions are robust in the system.

RBS is the starting site of the sequence in which mRNA is translated into protein. The efficiency of RBS may vary, depending on the binding strength (*k*_*r*_) of different sequences to the ribosome, which affects the protein yield. Changing the RBS-binding strength is the most feasible method for the synthetic circuit to control population density among the three parameters. Therefore, we quantitatively analyze how the RBS-binding strength of *ccdB* gene (*k*_*r*_) controls population density upon different IPTG induction levels. As indicated in Fig. 5b, the increase of RBS-binding strength hardly regulates population density upon low-dose IPTG induction (~0.1 to ~20 μM). However, population density can be regulated by RBS-binding strength at a wide range (i.e., 0.1 < *k*_*r*_ < 10), but within a narrow IPTG-induction range (i.e., ~20 to ~90 μM). Moreover, for efficient control, the binding strength should be restricted in a weak-strength range (i.e., 0.1 < *k*_*r*_ < ~0.3) with high-dose IPTG induction from ~100 to ~1000 μM.

We further systemically study the change of bacteria-growth curve under different RBS-binding strengths upon 1000 μM IPTG induction (Fig. 5c). Unexpectedly, we find that the bacterial growth curve begins to exhibit a double-peak pattern when the RBS efficiency is at the strength about 0.3. As the RBS efficiency increases, the double-peak phenomenon on the growth curve becomes more obvious. The growth curves that correspond to three typical binding strengths (i.e., *k*_*r*_ = 0.4, 0.5, and 0.6) are shown in the right panel of Fig. 5c. However, the second peak disappears again with the strength around 0.6.

Based on the above results, we suggest that the emergence of the only double-peak growth curve is due to the bacterial death induced by toxic metabolic by-products. We therefore hypothesize that an oscillation behavior of the bacterial growth curve might be occurring under certain conditions. Dynamic behavior changes of the growth curve with different toxic metabolic coefficients (i.e., *a*) are evaluated with the RBS-binding strength at 0.6. As the result suggests in Fig. 5d, oscillatory behavior of the bacterial growth curve is observed when the coefficient is smaller than ~0.02 min^−1^. With the decrease in the toxic metabolic coefficients, the oscillation behavior becomes stronger, giving increased oscillation amplitude and frequency. The four typical growth curves shown in the right panel of Fig. 5d indicate the behavior of double-peak changes to oscillation when the coefficient changes from 0.04 min^−1^ (black line) to 0.01 min^−1^ (red line). The oscillation amplitude and frequency significantly increase with decreasing the coefficient to 0.001 min^−1^ (blue line). Further decreasing the coefficient (*a* = 0.0001 min^−1^) barely influences the oscillation behavior (green line). Hence, the above analysis suggested that the toxic metabolic by-products can largely limit the oscillation behavior of bacteria-growth curve.

### Oscillation mechanism induced by the negative feedback loop—from the bifurcation viewpoint

**Fig. 6:**
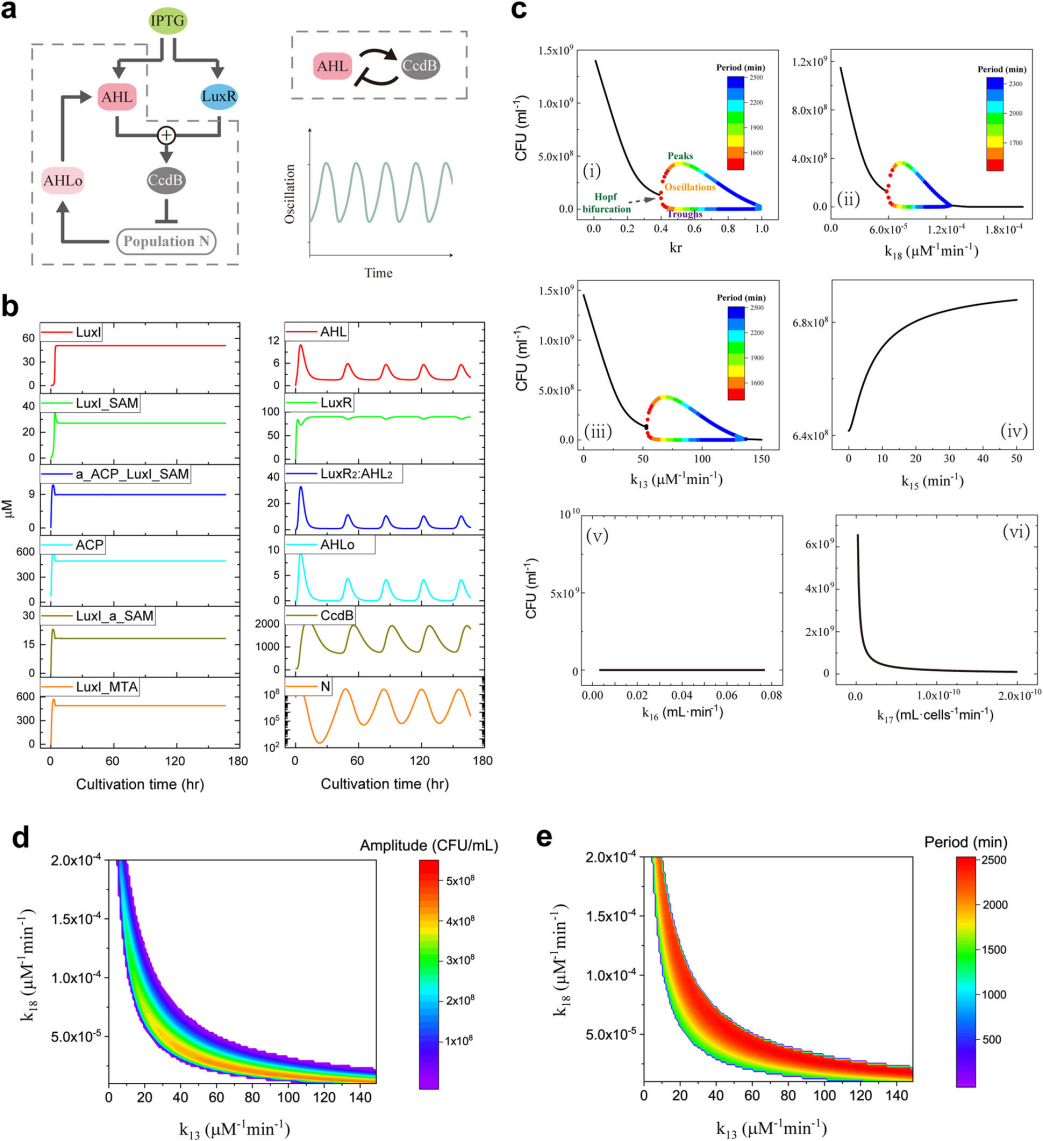
Mechanism analysis of oscillation in the quorum sensing system. **a** Schematic diagram of the negative feedback loop structure in the quorum sensing system. **b** Time courses of the key components in the two major parts shown in Fig. 1. **c** Bifurcation diagrams of the six represented parameters in modulating bacterial density. **d**–**e** Two-parameter bifurcation diagrams of the amplitude and period as a function of *k*_13_ and *k*_18_. Oscillation occurs within the color region and no oscillation occurs in the white region.

Oscillation state of quorum sensing is more important than the steady state in the process of bacterial delivery of drugs^15,40^. We next seek to systemically determine the mechanism underlying of the emergence of oscillation. As shown in Fig. 1, the quorum sensing signaling can be roughly divided into two parts: the left part of the loop circuit-induced AHL synthesis, and the right part of the AHL-triggered cell death. Actually, a negative feedback loop structure is included in the right part. As the simplified structure is shown in Fig. 6a, IPTG induces the expression of AHL and LuxR, which subsequently bind together to induce the expression of CcdB. CcdB inactivates DNA and triggers cell death. The concentration of AHL outside the bacteria is certainly increased with the increase of population density, positively regulating the AHL concentration inside the bacteria. Hence, a negative feedback loop structure between AHL and CcdB appeared. AHL promotes the expression of CcdB, while CcdB negatively regulates AHL through triggering cell death. In fact, the negative feedback loop is a well-identified mechanism to produce oscillatory dynamics^41^.

We further examine whether the oscillation is indeed induced by the negative feedback loop. Kinetics of the key components in the two parts of the quorum sensing signaling are presented in Fig. 6b. For the bacterial density that exhibits oscillation, oscillatory behavior occurs in the right part of the AHL-triggered cell death in Fig. 1 (Fig. 6b, right panel). While the left part of the loop circuit-induced AHL synthesis reaches stability (Fig. 6b, left panel). Hence, only the part that includes the negative feedback loop exhibits oscillatory behavior in the quorum sensing signaling.

Bifurcation analysis is employed to explore the emergence of the periodic oscillation. Since the toxic factors largely limit the oscillation behavior (Fig. 5d), the toxic metabolic coefficient (parameter a) is fixed at 0. Bifurcation diagram of cell density with *k*_*r*_, which is a key factor involved in the negative feedback structure, is shown in Fig. 6ci. The solid black lines represent the stable equilibrium points of the system. The colored lines represent the maximum and minimum amplitudes of the oscillations and the colors of the dots indicate the periods. Figure 6ci shows that when *k*_*r*_ is small, the steady state of the bacterial density is decreased with the increase of *k*_*r*_, but when *k*_*r*_ is beyond a value about 0.4, the bacterial density presents oscillatory behaviors. The oscillation amplitude is first increased and then decreased with the increase of *k*_*r*_, while the period keeps increasing. Besides, bifurcation analysis of other five typical parameters is studied as well (Fig. 6cii–cvi). The results of the other three parameters within the negative feedback structure, i.e., AHL-induced expression of CcdB (*k*_13_), CcdB negatively regulated bacterial density (*k*_18_), and AHL flowing out of bacteria (*k*_15_), are shown in Fig. 6cii–civ. Both the parameters of *k*_13_ and *k*_18_ can induce the occurrence of oscillation and exhibit dynamical behavior similar to *k*_*r*_ (Fig. 6cii, ciii). The oscillation ranges for *k*_13_ and *k*_18_, respectively, corresponding to the strengths of about 50–140 μM^−1^ min^−1^ and 6 × 10^−5^−1.2 × 10^−4^ μM^−1 ^min^−1^. While the steady state of density is increased with the increase of *k*_15_ (Fig. 6civ), indicating that high AHL permeability results in high steady states of bacterial density. The results of the two parameters (*k*_16_ and *k*_17_) that directly mediate bacterial density but are not included in the negative feedback structure suggest that oscillation cannot be triggered by these two corresponding reactions (Fig. 6cv, cvi).

A more holistic view on the dynamic behavior of the system can be characterized by the phase diagram when two reaction strengths are varied simultaneously. The two-parameter bifurcation diagram based on *k*_13_ and *k*_18_ suggests that oscillation can be obtained in a broad parameter range (Fig. 6d). When *k*_13_ is small, oscillations occur with a strong strength of *k*_18_. While when *k*_18_ is small, *k*_13_ needs to be large for inducing oscillations. For a given strength of *k*_13_, increase of *k*_18_ seems to reduce the amplitude (Fig. 6d), and enlarges the period (Fig. 6e). Therefore, the above results unravel the mechanism underlying the emergence of periodic oscillation and provide a quantitative overview of how the corresponding reaction strengths shift the dynamical behaviors of the quorum sensing system.

### Two faces of the toxic metabolic factors in controlling oscillatory behavior—from the global stability viewpoint

**Fig. 7:**
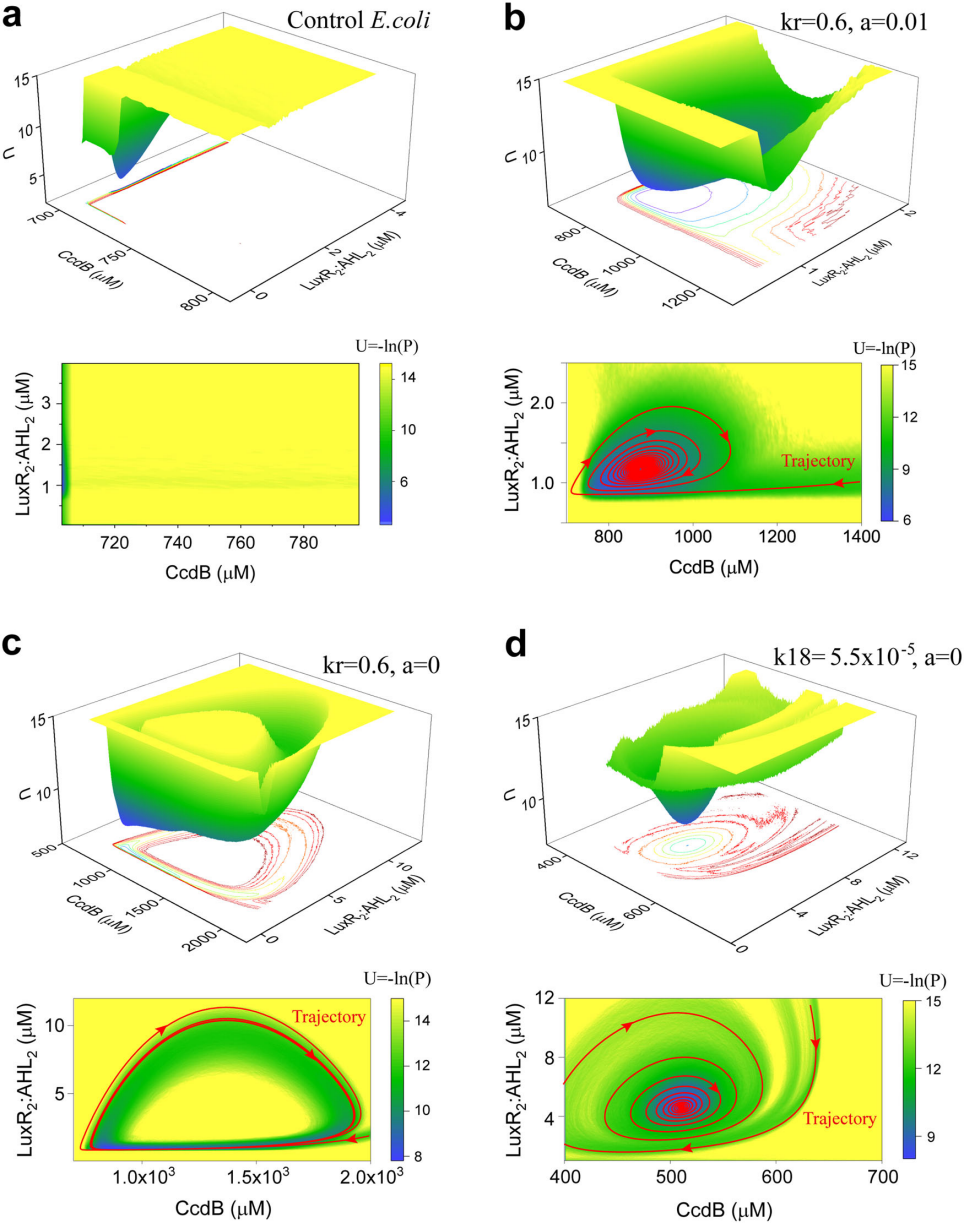
Landscape changes when the oscillation-induction parameters and toxic coefficient are varied. The control *E.coli* system exhibits a monostable landscape (**a**). Variations of *k*_*r*_ and the toxic coefficient (*a*) switch the system to the vortex-like pattern landscape (**b**), whereas the system presents the Mexican hat landscape after removing the effect of toxic factors (**c**). Variations of *k*_18_ and a can also switch the system to the vortex-like pattern landscape (**d**).

To systematically study the stochastic properties of the quorum sensing system oscillation, we further employ the recently developed potential landscape theory^42–44^ that describes the global dynamic behavior of the system in phase space. The dimensionless potential (*U*) and steady-state probability distribution (*P*) of the system is given by the Boltzmann relation, that is, *U* = −ln(*P*). For the control *E.coli* system, the corresponding potential landscape that mapped onto the CcdB–LuxR_2_:AHL_2_ phase space is shown in Fig. 7a. As a result, the system exhibits a monostable landscape (a stable steady state corresponding to low cell density), implying that the system evolves into a unique state from any initial values. The yellow region represents higher potential or lower probability, and the blue region represents lower potential or higher probability.

To investigate the influence of the oscillation-induction reactions on the potential landscape, we show the change of landscape when the reaction strength is varied. When respectively fixing the RBS-binding strength (*k*_*r*_) and the toxic metabolic coefficient (*a*) at 0.6 and 0.01, the system presents a monostable basin with a much larger area (Fig. 7b, upper panel). Different from the landscape shown in Fig. 7a, the trajectory of the initial values evolves into the unique state like a vortex in the landscape shown in Fig. 7b. The phase diagram indicates that the system presents a damped oscillation behavior (Fig. 7b, lower panel). Taking a typical evolution trajectory as an example, the system eventually evolves into the vortex-like pattern from any initial states.

The toxic metabolic coefficient, *a*, is identified to block the emergence of the periodic oscillation (Fig. 5d). We next set *a* = 0 to evaluate the change of the landscape topography. The system goes through a transition from the monostable basin to the Mexican hat landscape (Fig. 7c, upper panel), indicating that the system switches to a robust oscillation stage. The characteristics of the oscillation behavior can be displayed globally by quantifying the landscape topography. The size of the potential well quantitatively characterizes the amplitude, while the depth and breadth of the potential well reflect the stability and attraction domain of the limit-cycle attractor. As the evolution trajectory is shown in the phase diagram, the system eventually evolves into a stable limit cycle from any initial states (Fig. 7c, lower panel). Besides *k*_*r*_, we further evaluate the parameter of *k*_18_, which is also important for inducing oscillations within the negative feedback structure (Fig. 6cii). Similar to *k*_*r*_, variation of *k*_18_ can also switch the system to the vortex-like landscape (Fig. 7d), and further increase of *k*_18_ will induce the emergence of Mexican hat landscape with robust oscillation.

**Fig. 8:**
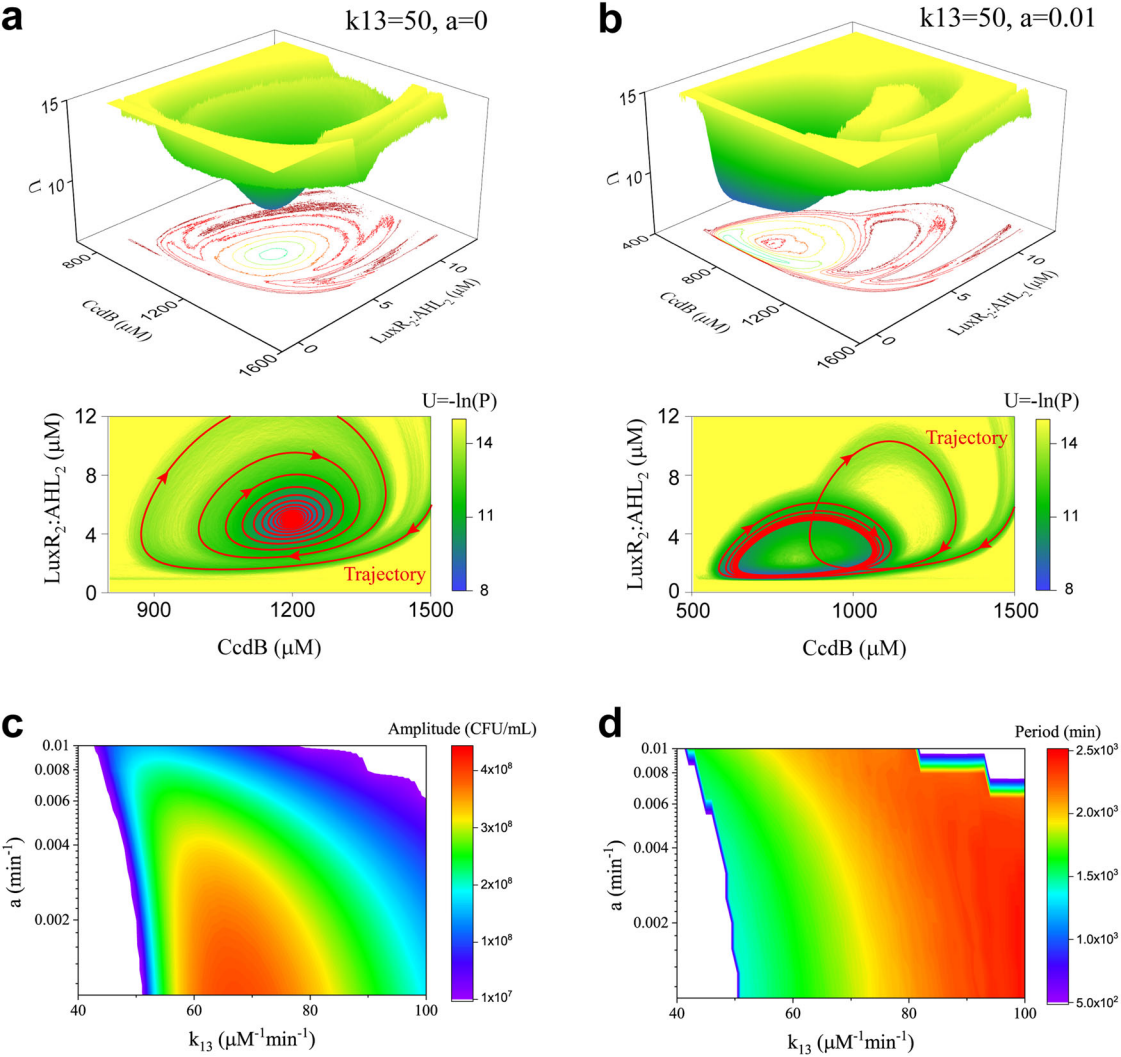
Dual roles of toxic metabolic factors in controlling oscillation in the quorum sensing system. **a**, **b** Addition of the toxic metabolic factors switches the system from the vortex-like pattern landscape to the Mexican hat landscape. **c**, **d** Two-parameter bifurcation diagrams of the amplitude and period as a function of *k*_13_ and *a*. Oscillations occur within the color region and no oscillation occurs in the white region.

In the previous section, we find that the toxic metabolic coefficient (*a*) suppresses the oscillatory behavior of bacteria-growth curve (Fig. 5d). The conclusion can also be obtained through comparing the landscapes shown in Fig. 7b, c. However, unexpected results are observed. The system presents the vortex-like landscape with damped oscillation behavior when *k*_13_ = 50 and *a* = 0 (Fig. 8a). While when we set *a* = 0.01, the Mexican hat landscape with robust oscillation behavior emerges (Fig. 8b), indicating that the introduced effect of toxic metabolic factors facilitates the occurrence of oscillatory behavior. Therefore, the toxic metabolic factors have two faces, which can both limit and promote the induction of oscillation in the quorum sensing system.

To quantify the dual roles of the toxic metabolic factors for oscillation induction, the two-parameter bifurcation diagram of the toxic coefficient (parameter *a*) and *k*_13_ is investigated. As the phase diagram suggested (Fig. 8c), the toxic factors promote the induction of oscillation at weak reaction strengths of the CcdB expression induced by LuxR_2_:AHL_2_ (*k*_13_ < 50 μM^−1^ min^−1^), while limit oscillation at strong strengths (*k*_13_ > 50 μM^−1^ min^−1^). Thus, 50 μM^−1^ min^−1^ of *k*_13_ is an important cut-off strength for discriminating the roles of toxic factors. The result also indicates that the amplitude is increased with the increase of toxic coefficient at a small *k*_13_, but is decreased at a large *k*_13_. However, the period is increased with the increase of toxic coefficient only at the range of approximately 55 μM^−1^ min^−1^ < *k*_13_ < 75 μM^−1^ min^−1^, but is barely influenced at other ranges (Fig. 8d). For the other two reaction parameters, *k*_*r*_ and *k*_18_, we have similar conclusions.

**Fig. 9:**
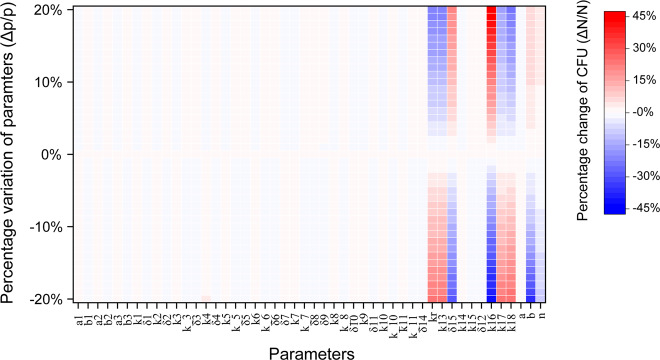
Sensitivity analysis of all the model parameters. The two-dimensional heatmap represents the corresponding percentage variation of the bacterial density to the percentage change (from −20% to 20% of the standard value) for each kinetic parameter.

Since the experimental data are not sufficient to fit all the parameters in our model, the choice of some parameter values is to some extent arbitrary. Sensitivity analysis of all the model parameters is therefore performed to evaluate the robustness of the parameter-dependent bacterial density. Similar to the result shown in Fig. 5a, the bacterial density is only sensitive to a few parameters (Fig. 9). More specifically, the density is insensitive to the upstream signaling parameters, but can be only efficiently controlled by a few downstream parameters. Despite that the upstream experimental data are lacking for fitting, Fig. 9 indicates that the upstream signaling parameters are rather robust to bacterial density and the parameter selection has a limited influence on it. Thus, the proposed optimal control strategy and the identified underlying mechanism for oscillation remain valid.

## Discussion

Bacteria use quorum sensing to sense changes in concentration and the surrounding environment, thereby regulating the expression of related genes and physiological activities for better adapting to environment variations^45–47^. In this paper, we use experimental data to develop a mathematical model of a synthetic quorum sensing system in *E. coli*, with the aim of providing a quantitative picture of the circuit to quantify the optimal strategy for controlling bacterial population density. Simulation results are in good agreement with experimental data, particularly for the case of growth curves with different RBS-binding efficiency during the stationary phase. Using the constructed model, dynamical behaviors of the key proteins, complexes and the promoter responding probabilities are systemically studied, which are difficult to be explored in experiments at present. Compared with previous studies, several new insights are provided by this study on the system, such as how the components, i.e., IPTG, AHL permeability, and RBS efficiency, mediate the bacterial density. The RBS efficiency-induced damping oscillation behavior is predicted as well. We further uncover that the oscillatory behaviors are determined by the included negative feedback loop structure in the downstream signaling of the quorum sensing. Moreover, based on the bifurcation diagram and landscape, the two faces of the toxic metabolic factors in controlling oscillation are quantified, which provides a physical and quantitative explanation for the dynamics switching of the bacterial density.

Although IPTG is widely used and functions as an efficient inducer in *E. coli* system, IPTG is not an innocuous inducer, which is potential toxicity to cause appreciable damage to *E. coli* with a high dose. Thus, for safely and efficiently controlling population density, the induction dose of IPTG should be low but with a regulatory function. Our model analysis captures the detailed response processes upon various IPTG-dose inductions and indicates that the optimal dose range of IPTG induction is about 10–500 μM for efficiently controlling population density of the synthetic quorum sensing system in *E. coli*. Thus, induction with 1000 μM IPTG stimulation might have toxic effects in the previous study. The results can be used to guide the development of more rational IPTG treatments for *E. coli*. However, the optimal range of IPTG is defined as the most sensitive range of population density to IPTG, which is obtained with the model that does not consider the IPTG toxicity in this study. A more accurate optimal nontoxic range would be further determined when considering the heterologous expression of protein-induced toxicity in our future work.

As an essential signaling molecule, AHL has been involved in various synthetic quorum sensing systems. Numbers of modeling studies that combined with experiments focused on exploring the complex dynamic processes of AHL^48^. Nilsson et al. previously studied the concentration changes of AHL inside the cell and in the biofilm over time with growth rate, diffusion of AHL, and autoinduction rate, suggesting that AHL-mediated phenotype can occur at a relatively low cell density and low concentration of external AHL^38^. Besides, You et al. determined that the lethal protein-production rate is restricted by the synthesis of AHL. The cell density at a stable steady state is proportional to the degradation rate of AHL^17^. In this work, we discuss AHL permeability and report that the bacteria population density can be regulated by the AHL permeability during the stationary phase. We find that the control of population density by AHL permeability is IPTG-dose dependent, and the controllable area of AHL upon different IPTG induction levels is systematically quantified. This analysis helped to refine and resolve the quantitative issues of elaborating the role of AHL in the efficient population control, which could be tested by further experiments. We notice that targeting the membrane and specific transport qualities of AHL with tailored transport are important issues in the quorum sensing system. In a very recent paper, Billot et al. reviewed the current engineering studies that targeted AHL-interfering enzymes for bacterial control^49^. The enzymatic engineering approaches might allow the development of a specific strategy for controlling AHL permeability experimentally in the future. Moreover, Lee et al. developed a system that disrupting quorum sensing signal molecules by using nanobiocatalysts regulates the physiological state of bacterial cells, resulting in the alleviation of biofilm formation^50^. Such a method might also be effectively employed to mediate AHL permeability. We hope our predictions can be tested by the future experiments with these novel techniques. However, our main focus in this study is to systematically analyze the dynamical behaviors of the components in the system. We hope to refine our model and thus to address these questions with further experimental observations in our future work.

Emerging evidence has suggested the important role of quorum sensing oscillatory behavior in drug delivery, which can present a regular interval of release with proper amplitude and period^15,40^. To fully understand the effect of oscillatory behavior on quorum sensing, an increasing number of theoretical studies have recently sprung up. Chen et al. found that the time delay in protein synthesis can induce the stability and oscillation of quorum sensing^51^. In addition, Wang and Tang provided a physical explanation of oscillations in terms of energy-driven processes in several cellular quorum sensing systems^52^. Nevertheless, our results show that, for the first time to our knowledge, the RBS-binding strength determines the oscillatory behavior of the population density, which is greatly restricted by the bacterial death induced by the toxic metabolic by-products. We quantify the RBS-strength range and the toxic metabolic coefficient range that responds for inducing the oscillation of population density. Different from the previously proposed time-delay mechanism^51^, we show that the negative feedback loop within the quorum sensing signaling is also an important regulatory mechanism for oscillation, which can efficiently induce the diverse oscillation amplitude and period (Fig. 6c, d). Besides the RBS (*k*_*r*_), reactions of the AHL-induced expression of CcdB (*k*_13_) and CcdB negative-regulated cell density (*k*_18_) are also identified to be essential for oscillation and the efficient controllable strength ranges are quantified through bifurcation analysis. The amplitude and period in the bacterial population control are important for the drug delivery, which can determine the dose and time of the drug released by the quorum sensing system, respectively. Experimental studies suggested that the oscillatory amplitude and period of quorum sensing system are essential for treating the diseases that require periodic dosing of drugs, such as diabetes, high blood pressure, and cancer^15,53^. The regulatory mechanism of oscillation discussed in the paper might provide useful guidance for the drug delivery. Moreover, the oscillatory amplitude and period of bacterial population might be also important for division-site selection, circadian clocks, and wastewater treatment^54,55^.

Compared with the previous studies on quorum sensing modeling that most focused on the deterministic dynamics, our study from the landscape viewpoint quantitatively provides the stochastic dynamics, the global nature, and the kinetic transitions of the quorum sensing system. Although noise can drive many biological processes^56,57^, our stochastic dynamics analysis of the quorum sensing system indicates that the vortex-like landscape or Mexican hat landscape of the system is barely influenced by the small stochastic fluctuations. The landscape topography presents more dynamical properties of the system, such as the damped oscillation behavior that cannot be observed by the bifurcation analysis. Using this approach, we further identify the dual roles of the toxic metabolic factors in regulating oscillation, which have not been reported so far. Although the toxic effects are not included in the negative feedback loop structure, the toxic factors can also trigger oscillation through mediating the bacterial density. In the quorum sensing system, the toxic factors negatively regulate cell density. As the quantified range of the toxic factors for inducing oscillation is shown in Fig. 8c, when the reaction strength of the AHL-induced expression of CcdB (*k*_13_) is strong, a high density is required for oscillation. Thus, the reaction strength of the toxic factors (*a*) tends to be weak to reduce its negative effect on cell density. While when *k*_13_ is small, the toxic coefficient (*a*) should be large to decrease the cell density for efficient oscillation. Overall, these results present new insights into the control mechanism of quorum sensing system and provide possible clues for biological implications.

As a fact, the lacking of experimental validations is a limitation of this modeling study. Nevertheless, our model accurately integrates the known biochemical reactions and can well reproduce the observed experimental data. Experimental data-based modeling approach is widely employed for dynamic discussion in various quorum sensing systems^19,58,59^. Motivated by the roles of AHL permeability and RBS efficiency that are still unclear, we thus developed the model to systematically evaluate its functions in the quorum sensing system and hope that our predictions can be tested by experiment in the future.

Despite lacking experimental validations, several previous experimental observations qualitatively support our predictions. We predicted that the optimal IPTG-induction range is between 10 and 500 μM, which is consistent with the experimental observation that a recombinant E. coli system shows increasing response with increasing IPTG dose up to 400 μM^60^. Since changing the AHL permeability is difficult in experiments, no experimental tests are done currently. However, two previous experimental studies have explored the combination effects of AHL and IPTG concentration on the dynamical response of the artificial E. coli system^35,36^. The two experimental observations show that the system presents efficient responses for both the AHL and IPTG concentrations at high levels. Actually, in our model, high AHL permeability will induce the high level of AHL outside the bacteria, which essentially has the same effects on the system as the two experimental studies. Thus, our prediction of the AHL permeability can be indirectly validated by these two studies. Moreover, a previous experimental study found that deletion of RBS abolishes oscillation behavior of *E. coli* cell density, proving that RBS is essential for inducing oscillation^61^. Besides, Elowitz and Leibler determined that bacterial oscillations are favored by efficient RBS strengths^62^. Both of the two experimental researches can qualitatively support our prediction of the RBS-induced oscillation. Compared with these studies, our study further quantitatively explored the relationship between RBS-binding strengths and the oscillatory behavior.

Despite specific to an exact gene circuit, our conclusion of the functional roles of components might be generally applicable. This study not only advances our knowledge of various regulatory mechanisms of this circuit, but also provides a representative framework to systematically study the underlying mechanisms in other synthetic circuits, ultimately providing potential clues for the development of more rational control strategy for various quorum sensing systems^20,63^. With the combination of further experimental observations, we hope to propose a more comprehensive model to make up the shortages, e.g., the simplification of by-product-induced bacterial death, specific to an exact gene circuit, lacking experimental validation and so on, thus providing more convincing results in our future work.

## Methods

### Model construction of the quorum sensing system

Ordinary differential equation (ODE)-based modeling is a well-established approach and has been widely used to quantitatively study the cellular-regulatory mechanism^64,65^. The bacteria state can be described by the component concentrations (*C*_1_, *C*_2_,…). Based on the law of mass action, the reaction rates are dependent on these concentrations and the kinetic parameters (*k*_1_, *k*_2_,…). The model is formulated as a set of coupled ODEs to describe the time evolution of component concentrations in terms of the following general equation:

1 $$\frac{{{\mathrm{d}}C_i}}{{{\mathrm{d}}t}} = \mathop {\sum}\limits_{j = 1}^n {v_{ij} \cdot q_j,\left( {i = 1,...,m} \right)}$$

where: d*C*_*i*_/d*t* is the concentration-changing rate of component *i* with time; *m* represents the number of components with the concentration *C*_*i*_; *n* is the number of reactions with the rate *q*_*j*_; and *v*_*ij*_ denotes the element of the stoichiometric matrix^66^ that links the reaction rates of *q*_*i*_ with component *C*_*i*_. Complete description of ODEs in a model that describes the dynamics of the component in different modules is presented below. The ODE model is developed and simulated with MATLAB and the ode 15s function of MATLAB is used to solve ODEs^67^. The zipped source code file can be found in https://github.com/jianweishuai/QS.

### Module of gene transcription

Each promoter of the corresponding genes has two states, i.e., the promoter that responds to the inducer and the promoter that does not respond to the inducer. The probabilities of the *luxI* gene, *luxR* gene, and *ccdB* gene promoter responding to the inducer are represented by *P*_I1_, *P*_R1_, and *P*_B1_, respectively. Because a promoter has only two states, the sum of the probability of these two states is 1. Then, 1–*P*_I1_, 1–*P*_R1_, and 1–*P*_B1_ represent the probability of three promoters non-responding to the inducer, respectively. In our model, the transcription processes of the three genes are independent to each other. We assume there is not any limited molecular mechanism during the expression of the three genes. Compared with the changes in protein levels, the transcription factor activity presents the dynamics on the fast timescale^68^. We therefore consider the transcription processes to be at a steady state without explicitly modeling^69^.

Thus, the kinetic equations of the probabilities of IPTG inducing the transcription of the *luxI* gene and *luxR* gene, and the probability of LuxR_2_:AHL_2_ complex inducing the transcription of the *ccdB* gene, are

2 $$\frac{{{\mathrm{d}}P_{I1}}}{{{\mathrm{d}}t}} = a_1\left( {1 - P_{I1}} \right)\left[ {{\mathrm{IPTG}}} \right] - b_1P_{I1}$$

3 $$\frac{{{\mathrm{d}}P_{R1}}}{{{\mathrm{d}}t}} = a_2\left( {1 - P_{R1}} \right)\left[ {{\mathrm{IPTG}}} \right] - b_2P_{R1}$$

4 $$\frac{{{\mathrm{d}}P_{B1}}}{{{\mathrm{d}}t}} = a_3\left( {1 - P_{B1}} \right)\left[ {{\mathrm{LuxR}}_2:{\mathrm{AHL}}_2} \right] - b_3P_{B1}$$

The biochemical reaction of LuxI degradation, LuxI:SAM association/disassociation, and the reaction of LuxI:MTA complex association/disassociation are described by the following:

$${\mathrm{LuxI}}\,\mathop { \to }\limits^{\delta _1} \,\Phi ;{\mathrm{LuxI}} + {\mathrm{SAM}}\,\mathop { \leftrightarrow }\limits_{k_{ - 3}}^{k_3} \,{\mathrm{LuxI:SAM;LuxI:MTA}}\,\mathop { \leftrightarrow }\limits_{k_{ - 8}}^{k_8} \,{\mathrm{LuxI}} + {\mathrm{SAM}}$$

Thus, dynamic behavior of LuxI protein can be represented by the equation

5 $$\begin{array}{l}\frac{{{\mathrm{d}}\left[ {{\mathrm{LuxI}}} \right]}}{{{\mathrm{d}}t}} = k_1P_{I1} - \delta _1\left[ {{\mathrm{LuxI}}} \right] - \left( {k_3[{\mathrm{LuxI}}][{\mathrm{SAM}}] - k_{ - 3}[{\mathrm{LuxI:SAM}}]} \right)\\ \qquad\qquad+ \,k_8\left[ {{\mathrm{LuxI:MTA}}} \right] - k_{ - 8}\left[ {{\mathrm{LuxI}}} \right]\left[ {{\mathrm{MTA}}} \right]\end{array}$$

The biochemical reactions of LuxR degradation and LuxR:AHL complex association/disassociation are

$${\mathrm{LuxR}}\,\mathop { \to }\limits^{\delta _{11}} \,\Phi ;{\mathrm{LuxR}} + {\mathrm{AHL}}\,\mathop { \leftrightarrow }\limits_{k_{ - 11}}^{k_{11}}\, {\mathrm{LuxR:AHL}}$$

The kinetic equation of LuxR protein is

6 $$\frac{{{\mathrm{d}}\left[ {{\mathrm{LuxR}}} \right]}}{{{\mathrm{d}}t}} = k_9P_{R1} - \delta _{11}\left[ {{\mathrm{LuxR}}} \right] - \left( {k_{11}\left[ {{\mathrm{LuxR}}} \right]\left[ {{\mathrm{AHL}}} \right] - k_{ - 11}\left[ {{\mathrm{LuxR:AHL}}} \right]} \right)$$

Dynamical behavior of the killer protein CcdB, which involves the processes of LuxR_2_:AHL_2_-complex-induced production of CcdB protein, and the wild-type CcdB protein production and degradation, is described by the following equation:

7 $$\frac{{{\mathrm{d}}\left[ {{\mathrm{CcdB}}} \right]}}{{{\mathrm{d}}t}} = k_rk_{13}P_{B1} + k_{14} - \delta _{15}\left[ {{\mathrm{CcdB}}} \right]$$

RBS is the starting site of the sequence in which mRNA is translated into protein. The efficiency of RBS varies depending on the binding strength (*k*_*r*_) of different sequences to the ribosome, which affects the protein yield. The parameter *k*_13_ represents the intrinsic production rate of CcdB by the synthesis gene. Since the CcdB production depends on the binding strength of RBS, we thus multiply the two parameters, *k*_*r*_ and *k*_13_ together in the first term.

### Module of AHL synthesis

The reaction processes of LuxI protein use SAM and acyl-ACP as substrates to synthesize AHL mainly containing the processes of LuxI:a_SAM complex association/disassociation (a_SAM denotes the activated form of SAM in complex), dimer AHL_2_ aggregation/disaggregation, degradation of AHL, and AHL flows out of bacteria. The biochemical reactions are accordingly presented below:

$${\mathrm{LuxI}}:{\mathrm{a}}\_{\mathrm{SAM}}\,\mathop { \leftrightarrow }\limits_{k_{ - 7}}^{k_7} \,{\mathrm{LuxI}}:{\mathrm{MTA}} + {\mathrm{AHL;AHL}}\,\mathop { \leftrightarrow }\limits^{k_{15}} \,{\mathrm{AHL}}_{\mathrm{O}}$$

Thus, the dynamic change of AHL is described by the following equations:

8 $$\begin{array}{l}\frac{{{\mathrm{d}}\left[ {{\mathrm{AHL}}} \right]}}{{{\mathrm{d}}t}} = k_7\left[ {{\mathrm{LuxI}}:{\mathrm{a}}\_{\mathrm{SAM}}} \right] - k_{ - 7}\left[ {{\mathrm{AHL}}} \right]\left[ {{\mathrm{LuxI}}:{\mathrm{MTA}}} \right]\\ \qquad\qquad- \left( \begin{array}{l}k_{11}\left[ {{\mathrm{LuxR}}} \right]\left[ {{\mathrm{AHL}}} \right]\\ - k_{ - 11}\left[ {{\mathrm{LxuR}}:{\mathrm{AHL}}} \right]\end{array} \right) - \delta _9\left[ {{\mathrm{AHL}}} \right] - k_{15}\left( {\left[ {{\mathrm{AHL}}} \right] - \left[ {{\mathrm{AHL}}_{\mathrm{O}}} \right]} \right)\end{array}$$

Besides the AHL inside the bacteria, AHL outside the bacteria is also an important factor. The dynamics of the outside AHL_o_ can be described by the following equation:

9 $$\frac{{{\mathrm{d}}\left[ {{\mathrm{AHL}}_{\mathrm{O}}} \right]}}{{{\mathrm{d}}t}} = k_{15}\left( {\left[ {{\mathrm{AHL}}} \right] - \left[ {{\mathrm{AHL}}_{\mathrm{O}}} \right]} \right)\frac{{NV_1}}{{V - NV_1}} - \delta _{12}\left[ {{\mathrm{AHL}}_{\mathrm{O}}} \right]$$

In Eq. (9), [AHL] – [AHL_o_], which represents the concentration difference of AHL between the inside and outside of the bacteria, is the driving force of AHL flowing out of bacteria. *N* is the population of bacteria. *V*_1_ is individual *E. coli* volume and *V* is the volume of the entire culture environment. The concentration of AHL flowing out of the bacteria is related to the volumes inside and outside of the bacteria. Thus, the volume ratio of the bacteria volume to the entire culture environment, $\frac{{{\it{NV}}_{\it{1}}}}{{{\it{V}} - {\it{NV}}_{\it{1}}}}$, has to be considered. The last term in the formula describes the degradation process of AHL_o_. The dynamics for other components in the AHL synthesis processes are described by the ODEs shown in Supplementary Table 1.

Biochemical reaction of the LuxR_2_:AHL_2_ tetramer formation by the combination of two LuxR:AHL complex is

$$2 \cdot \left[ {{\mathrm{LuxR}}:{\mathrm{AHL}}} \right]\,\mathop { \leftrightarrow }\limits_{k_{ - 10}}^{k_{10}} \,{\mathrm{LuxR}}_2:{\mathrm{AHL}}_2$$

The dynamics of the LuxR:AHL and LuxR_2_:AHL_2_ tetramer are described by the following equations:

10 $$\begin{array}{l}\frac{{{\mathrm{d}}\left[ {{\mathrm{LuxR}}_2:{\mathrm{AHL}}_2} \right]}}{{{\mathrm{d}}t}} = k_{10}\left[ {{\mathrm{LuxR}}:{\mathrm{AHL}}} \right]^2 - k_{ - 10}\left[ {{\mathrm{LuxR}}_2:{\mathrm{AHL}}_2} \right]\\ \qquad\qquad\qquad\quad- \delta _{14}\left[ {{\mathrm{LuxR}}_2:{\mathrm{AHL}}_2} \right]\\ \qquad\qquad\qquad\quad- \left( {a_3\left( {1 - P_{B1}} \right)\left[ {{\mathrm{LuxR}}_2:{\mathrm{AHL}}_2} \right] - b_3P_{B1}} \right)\end{array}$$

11 $$\begin{array}{l}\frac{{{\mathrm{d}}\left[ {{\mathrm{LuxR}}:{\mathrm{AHL}}} \right]}}{{{\mathrm{d}}t}} = k_{11}\left[ {{\mathrm{LuxR}}} \right]\left[ {{\mathrm{AHL}}} \right] - k_{ - 11}\left[ {{\mathrm{LuxR}}:{\mathrm{AHL}}} \right] \\ \qquad\qquad\qquad- \left( {k_{10}\left[ {{\mathrm{LuxR}}:{\mathrm{AHL}}} \right]^2 - k_{ - 10}\left[ {{\mathrm{LuxR}}_2:{\mathrm{AHL}}_2} \right]} \right)\end{array}$$

### Module of the bacteria population

The kinetic equation of bacteria population *N* is

12 $$\frac{{{\mathrm{d}}N}}{{{\mathrm{d}}t}} = k_{16}N - k_{17}N^2 - k_{18}\left[ {{\mathrm{CcdB}}} \right]N - \frac{{aNt^n}}{{t^n + b^n}}$$

We employ the logistic model to describe the general dynamics of bacterial proliferation and death^70^ represented by the first two terms shown in Eq. (12). The term *k*_16_*N* represents the natural increase, and *k*_17_*N*^2^ represents the decrease of cell population caused by the limited space and nutritional competition. The third term *k*_18_[CcdB]*N* describes the death induced by CcdB of the synthetic quorum sensing, which depends on the death rate *k*_18_, CcdB concentration, and cell population *N*. This formula is a general and simple treatment in biophysical modeling^70,71^. Since the effects of toxic metabolic by-products on cell death are complicated^72,73^, the Hill term *aNt*^*n*^/(*t*^*n*^ + *b*^*n*^) is therefore employed for simplification. During the early phase with a small *t*, a small amount of toxic metabolic by-products is produced, making little contribution to death. While during the later phase with a large *t*, the toxic metabolic by-products are accumulated massively, inducing cell death in a large population.

### Parameter-value determination

All the parameters are first restricted within the typical biological ranges according the reaction types^39,68^. Then we estimate the parameters based on the earlier literature. The parameters are chosen randomly within the typical biological ranges to avoid convergence to local minima. Then the parameters are further determined by a global optimization method that minimizes the deviation between simulation results and experiments of the bacteria-growth curves of wild-type *E. coli* and the engineered bacteria with RBS_0.07_ *E. coli*^21^, or consulted from previous literature. The deviation is characterized by using the correlation coefficient, *R*-square, which is determined as the following functions:

13 $$^{R^2 = 1 - \frac{{\mathop {\sum}\nolimits_{i = 1}^n {\left( {y_{\exp }\left( {t_i} \right) - y_{{\mathrm{sim}}}\left( {t_i} \right)} \right)^2} }}{{\mathop {\sum}\nolimits_{i = 1}^n {\left( {y_{\exp }\left( {t_i} \right) - \overline {y_{\exp }} } \right)^2} }}}$$

where *y*_exp_(*t*_*i*_) and *y*_sim_(*t*_*i*_) are the population density of the bacteria-growth curve data in experiment and simulation at time *t*_*i*_, respectively. Based on the previous experimental data^21^, the total volume of the culture is 1 mL in the model. The volume of an *E. coli* is about 1.6 μm^3^. The initial value of the bacteria population and the concentration of IPTG induction in the model are 4.47 × 10^7^ CFU/mL and 1000 μM. All the parameter descriptions, values, and units in the model are listed in Supplementary Table 2. The general qualitative behavior of the model is robust to small and even intermediate changes of most parameters, providing confidence that plausible parameter values are obtained (see details in Figs. 5a and 9).

### Parameter-sensitivity analysis

Parameter-sensitivity analysis is conducted to identify whether and how the parameters have influence on the bacteria population density during the stationary phase. Based on ref. ^74^, the local sensitivity coefficient is

14 $$S_{{\mathrm{local}}} = \frac{p}{N}\frac{{\Delta N}}{{\Delta p}}$$

where *p* is the parameter with Δ*p* the change of *p*, and *N* is the output with Δ*N* representing the change of population density. Each parameter *p* is varied ±20% from its default value and the corresponding percentage change of population density is calculated in the model accordingly.

### Identification of oscillation dynamics

The stable oscillation dynamics of the system is identified based on the previous study^75^, in which the oscillation coefficient is defined as follows:

15 $${\mathrm{OSC}} = \frac{{\left( {C_{{\mathrm{MAX}}} - C_{{\mathrm{MIN}}}} \right)}}{{\left( {C_{{\mathrm{MAX}}} + C_{{\mathrm{MIN}}}} \right)/2}}$$

where *C*_MAX_ and *C*_MIN_ represent the peak value and valley value of component concentration, respectively. The variable is recognized as oscillatory signaling when the value of the coefficient OSC is greater than 0.1. We investigate the evolutionary dynamics of the component over time and the peak values satisfy *C*_*t*_ *>* *C*_*t-1*_ and *C*_*t*_ *>* *C*_*t+1*_. If no less than 5 peaks are found in a long enough time (*t* from 100 h to 500 h), it can be identified as a stable oscillatory signaling. The amplitude of the oscillation is determined by calculating the difference between *C*_MAX_ and *C*_MIN_ within the stable oscillatory range, and the period is determined by calculating the time difference between two adjacent peak values.

### Potential landscape described by spatial density approximation

Based on the previous studies^42–44^, the stochastic dynamics of the continuous quorum sensing system is described by Langevin equation, i.e., d*C*_*i*_*(t)/*d*t* *=* *F(C*_*i*_*)* *+* *η(t)*, where *C*_*i*_ represents the concentration of the molecules or gene expression levels. The noise term *η(t)* adopts the independent additive white Gaussian noise, 〈*η*(*t*)〉 = 0 and 〈*η*(*t*)*η*(*t*ʹ)〉 = 2*Dδ*(*t* − *t*ʹ). To obtain the potential landscape of high-dimensional complex systems, it is obviously difficult to use Fokker–Planck equation to solve the evolution probability of systems in phase space. Starting from enough random initial conditions, the system will eventually evolve to a steady state, which may be monostable, multistable, or limit-cycle steady state. The evolution trajectory density near the attractor in the phase space is the highest. All evolution trajectories in high-dimensional phase space are mapped to any two-dimensional phase plane, and then the two-dimensional phase plane is divided into several small regions, and the trajectory density in each small region is counted. Using the distribution of trajectory density instead of the evolution-probability distribution of the system, the potential landscape of the system is obtained. In our study, the stochastic mathematical model adding Gaussian white noise (the intensity was fixed at 0.001) is simulated from random initial values for 10,000 times.

### Reporting summary

Further information on research design is available in the Nature Research Reporting Summary linked to this article.

## Supplementary information

Supplementary information.

Reporting summary.

## Data Availability

The models are developed and simulated with MATLAB R2020a and Python 3.70. The zipped source code file of the model to generate the results in this study can be found in https://github.com/jianweishuai/QS.
