# Supplementary material for: Quantifying the optimal strategy of population control of quorum sensing network in Escherichia coli
Source: NPJ Syst Biol Appl. 2021 Sep 2;7:35. doi: 10.1038/s41540-021-00196-4 (PMC8413372; doi:10.1038/s41540-021-00196-4)
Supplement: Supplementary file 1 — Supplementary information. [file 41540_2021_196_MOESM1_ESM.pdf]

Supplementary Files

Supplementary Table 1. Ordinary differential equations (ODEs) of the model.

$$dP_{I1}/dt = a_1\left(1-P_{I1}\right)\left[IPTG\right]-b_1P_{I1}$$

$$dP_{R1}/dt = a_2\left(1-P_{R1}\right)\left[IPTG\right]-b_2P_{R1}$$

$$dP_{B1}/dt = a_3\left(1-P_{B1}\right)\left[LuxR_2 : AHL_2\right]-b_3P_{B1}$$

$$d\left[LuxI\right]/dt = k_1P_{I1}-\delta_1\left[LuxI\right]-\left(k_3\left[LuxI\right]\left[SAM\right]-k_{-3}\left[LuxI:SAM\right]\right)$$
$$+k_8\left[LuxI:MTA\right]-k_{-8}\left[LuxI\right]\left[MTA\right]$$

$$d[LuxR]/dt = k_9P_{R1}-\delta_{11}[LuxR]-(k_{11}[LuxR][AHL]-k_{-11}[LuxR:AHL])$$

$$d[CcdB]/dt = k_rk_{13}P_{B1}+k_{14}-\delta_{15}[CcdB]$$

$$d\left[SAM\right]/dt = k_2-\delta_2\left[SAM\right]-\left(k_3\left[LuxI\right]\left[SAM\right]-k_{-3}\left[LuxI:SAM\right]\right)$$

$$d\left[LuxI:SAM\right]/dt = k_3\left[LuxI\right]\left[SAM\right]-k_{-3}\left[LuxI:SAM\right]-\delta_3\left[LuxI:SAM\right]$$
$$-\left(k_5\left[LuxI:SAM\right]\left[a\_ACP\right]-k_{-5}\left[a\_ACP:LuxI:SAM\right]\right)$$

$$d\left[a\_ACP\right]/dt = k_4-\delta_4\left[a\_ACP\right]-\left(k_5\left[LuxI:SAM\right]\left[a\_ACP\right]-k_{-5}\left[a\_ACP:LuxI:SAM\right]\right)$$

$$d\left[a\_ACP:LuxI:SAM\right]/dt = k_5\left[LuxI:SAM\right]\left[a\_ACP\right]-k_{-5}\left[a\_ACP:LuxI:SAM\right]$$
$$-\delta_5\left[a\_ACP:LuxI:SAM\right]-\left(k_6[a\_ACP:LuxI:SAM]-k_{-6}[ACP][LuxI:a\_SAM]\right)$$

$$d\left[ACP\right]/dt = k_6\left[a\_ACP:LuxI:SAM\right]-k_{-6}[ACP][LuxI:a\_SAM]-\delta_7[ACP]$$

$$d\left[LuxI:a\_SAM\right]/dt = k_6\left[a\_ACP:LuxI:SAM\right]-k_{-6}[ACP][LuxI:a\_SAM]$$
$$-\delta_6\left[LuxI:a\_SAM\right]-\left(k_7\left[LuxI:a\_SAM\right]-k_{-7}[AHL][LuxI:MTA]\right)$$

$$d[AHL]/dt = k_7[LuxI:a\_SAM]-k_{-7}[AHL][LuxI:MTA]-(k_{11}[LuxR][AHL]$$
$$-k_{-11}[LxuR:AHL])-\delta_9[AHL]-k_{15}([AHL]-[AHL_O])$$

$$d\left[LuxI:MTA\right]/dt = k_7\left[LuxI:a\_SAM\right]-k_{-7}[AHL][LuxI:MTA]$$
$$-\left(k_8[LuxI:MTA]-k_{-8}[LuxI][MTA]\right)-\delta_8[LuxI:MTA]$$

$$d\left[MTA\right]/dt = k_8\left[LuxI:MTA\right]-k_{-8}[LuxI][MTA]-\delta_{10}[MTA]$$

$$d[LuxR:AHL]/dt = k_{11}[LuxR][AHL]-k_{-11}[LuxR:AHL]-$$
$$(k_{10}[LuxR:AHL]^2-k_{-10}[LuxR_2:AHL_2])$$

$$d[LuxR_2:AHL_2]/dt = k_{10}[LuxR:AHL]^2-k_{-10}[LuxR_2:AHL_2]-\delta_{14}[LuxR_2:AHL_2]$$
$$-(a_3(1-P_{B1})[LuxR_2:AHL_2]-b_3P_{B1})$$

$$d\left[AHL_O\right]/dt = k_{15}\left(\left[AHL\right]-\left[AHL_O\right]\right)\cdot NV_1/\left(V-NV_1\right)-\delta_{12}\left[AHL_O\right]$$

$$dN/dt = k_{16}N-k_{17}NN-k_{18}[CcdB]N-aNt^n/(t^n+b^n)$$

Supplementary Table 2. Model parameter values, units, and descriptions.

Parameter	Value	Unit	Description	References
a_1	2.0e-5	min^{-1}	Responding rate of IPTG and P_{LuxI}	Chen et al.
b_1	4.5e-3	$\mu M \cdot min^{-1}$	Non-responding rate of IPTG and P_{LuxI}	Chen et al.
a_2	2.0e-5	min^{-1}	Responding rate of IPTG and P_{LuxR}	Chen et al.
b_2	4.5e-3	$\mu M \cdot min^{-1}$	Non-responding rate of IPTG and P_{LuxR}	Chen et al.
a_3	0.001	min^{-1}	Responding rate of $LuxR_2$:AHL_2 and P_{CcdB}	Chen et al.
b_3	0.003	$\mu M \cdot min^{-1}$	Non-responding rate of $LuxR_2$:AHL_2 and P_{CcdB}	Chen et al.
k_1	72.0	$\mu M \cdot min^{-1}$	Production rate of LuxI	You et al.
δ_1	0.16	min^{-1}	Degradation rate of LuxI	You et al.
k_2	51.0	$\mu M \cdot min^{-1}$	Production rate of SAM	Estimated
δ_2	0.01	min^{-1}	Degradation rate of SAM	Lee & Bailey
k_3	0.05	$\mu M^{-1} \cdot min^{-1}$	Association rate of LuxI and SAM	Estimated
k_{-3}	0.2	min^{-1}	Disassociation rate of LuxI and SAM	Estimated
δ_3	0.096	min^{-1}	Degradation rate of LuxI:SAM	Li et al.
k_4	50.0	$\mu M \cdot min^{-1}$	Production rate of a_ACP	Estimated
δ_4	0.01	min^{-1}	Degradation rate of a_ACP	Lee & Bailey
k_5	0.01	$\mu M^{-1} \cdot min^{-1}$	Association rate of LuxI:SAM and a_ACP	Estimated
k_{-5}	0.1	min^{-1}	Disassociation rate of LuxI:SAM and a_ACP	Estimated
δ_5	0.096	min^{-1}	Degradation rate of LuxI:SAM:a-ACP	Li et al.
k_6	5.8	min^{-1}	Production rate of ACP and LuxI:a_SAM	Fitted
k_{-6}	5.0e-4	$\mu M^{-1} \cdot min^{-1}$	Production rate of LuxI:SAM:a-ACP	Fitted
δ_6	9.6e-3	min^{-1}	Degradation rate of LuxI:a_SAM	Saeidi et al.
δ_7	0.096	min^{-1}	Degradation rate of ACP	Li et al.
k_7	2.6	min^{-1}	Production rate of AHL and LuxI:MTA	Fitted
k_{-7}	1.0e-4	$\mu M^{-1} \cdot min^{-1}$	Production rate of LuxI:a_SAM	Estimated
δ_8	0.096	min^{-1}	Degradation rate of LuxI:MTA	Estimated
δ_9	9.6e-5	min^{-1}	Degradation rate of AHL	You et al.
k_8	0.03	min^{-1}	Association rate of LuxI and MTA	Estimated

k_{-8}	0.2	$\mu M^{-1}\cdot min^{-1}$	Disassociation rate of LuxI and MTA	Estimated
δ_{10}	0.096	min^{-1}	Degradation rate of MTA	Li et al.
k_9	23	$\mu M\cdot min^{-1}$	Production rate of LuxR	You et al.
δ_{11}	0.039	min^{-1}	Degradation rate of LuxR	You et al.
k_{10}	0.172	$\mu M^{-1}\cdot min^{-1}$	Association rate of LuxR:AHL and LuxR:AHL	Estimated
k_{-10}	0.01	min^{-1}	Disassociation rate of $LuxR_2:AHL_2$	Lee & Bailey
k_{11}	0.005	$\mu M^{-1}\cdot min^{-1}$	Association rate of LuxR and AHL	You et al.
k_{-11}	0.543	min^{-1}	Disassociation rate of LuxR:AHL	You et al.
δ_{14}	0.096	min^{-1}	Degradation rate of $LuxR_2:AHL_2$	Li et al.
k_r	0.15	1	The binding strength of RBS	Fitted
k_{13}	20.0	$\mu M\cdot min^{-1}$	Production rate of CcdB by the synthesis gene	Estimated
δ_{15}	4.1e-3	min^{-1}	Degradation rate of CcdB	Fitted
k_{14}	0.22	$\mu M\cdot min^{-1}$	Production rate of CcdB by the wild gene	Fitted
k_{15}	30.0	min^{-1}	Permeability rate of AHL	Estimated
δ_{16}	4.6e-3	min^{-1}	Degradation rate of extracellular AHL	Fitted
k_{16}	0.031	$mL\cdot min^{-1}$	Basal reproduction rate of bacterial cell	Estimated
k_{17}	2.05e-11	$mL\cdot cells^{-1}\cdot min^{-1}$	Basal death rate of bacterial cell	Estimated
k_{18}	2.34e-5	$\mu M^{-1}\cdot min^{-1}$	Death rate of bacterial cell induced by CcdB	Fitted
a	0.04	min^{-1}	Toxic metabolic coefficients	Fitted
b	2200	min	Michaelis constant of toxic metabolic effect	Fitted
n	8	1	Hill coefficient of toxic metabolic effect	Estimated

Supplementary References:

Chen, L., Wang, R., Zhou, T., & Aihara, K. (2005). Noise-induced cooperative behavior in a multicell system. Bioinformatics, 21(11), 2722-2729.

Lee, S. B., & Bailey, J. E. (1984). Analysis of growth rate effects on productivity of recombinant Escherichia coli populations using molecular mechanism models. Biotechnology and bioengineering, 26(1), 66-73.

Saeidi, N., Arshath, M., Chang, M. W., & Poh, C. L. (2013). Characterization of a quorum sensing device for synthetic biology design: Experimental and modeling validation. Chemical Engineering Science, 103, 91-99.

Li, J., Wang, L., Hashimoto, Y., Tsao, C. Y., Wood, T. K., Valdes, J. J., et al. (2006). A stochastic model of Escherichia coli AI-2 quorum signal circuit reveals alternative synthesis pathways. Molecular systems biology, 2(1), 67.

You, L., Cox, R. S., Weiss, R., & Arnold, F. H. (2004). Programmed population control by cell–cell communication and regulated killing. Nature, 428(6985), 868-871.
